# Analysing gender disparities in youth sports coaching: an international survey (FEMCoach project)

**DOI:** 10.3389/fpsyg.2025.1560764

**Published:** 2025-05-07

**Authors:** Vanessa Dias, Julio Calleja-Gonzalez, Víctor López-Ros, Raquel Font-Lladó, Jorge Arede, Leonardo Cunha, Stella Douka, Bruno Rosa, Graça Pinto, Nuno Leite

**Affiliations:** ^1^Department of Sports Sciences, Exercise and Health, University of Trás-os-Montes and Alto Douro, Vila Real, Portugal; ^2^Department of Physical Education and Sports, Faculty of Education and Sport, University of the Basque Country (UPV/EHU), Vitoria-Gasteiz, Spain; ^3^Institute of Educational Research, University of Girona, Girona, Spain; ^4^Cape Verdean Olympic Committee, Praia, Cabo Verde; ^5^Department of Physical Education and Sport Science, Aristotle University of Thessaloniki, Thessaloniki, Greece; ^6^Associação para o Desenvolvimento do Desporto Jovem – ADDJ, Lisbon, Portugal; ^7^Research Centre in Sports Sciences, Health, and Human Development (CIDESD), University of Trás-os-Montes and Alto Douro, Vila Real, Portugal

**Keywords:** sports coaching, educational programmes, women, female, gender equality, youth sports

## Abstract

**Introduction:**

The issue of gender inequality has a significant impact on the sporting world, with a range of implications and consequences. In the field of coaching, women are confronted with inequity throughout their careers, which frequently has a detrimental impact. To gain a deeper insight into the under-representation of women in sport coaching, framing the Female Sport Coaching Training Programme (FEMCoach) project, an investigation was carried out to examine coaches' educational support. After reviewing the scientific literature, a survey was developed aiming to analyse the needs of women youth sports coaches in practice to design coaching educational programmes that promote gender equity in youth sports.

**Methods:**

The present study collected and analyzed survey data from 463 coaches (45% women and 55% men) from 24 different countries, sports and competitive levels. Coaches completed a 40-question online survey, divided into seven topics: (1) sociodemographic data, (2) the inclusion environment for sports practice, (3) coach professional activities, (4) menstrual cycle, hormonal contraception and pregnancy/childcare, (5) barriers for females in sport, (6) coaches' courses, and (7) gender differences.

**Results:**

Some differences were found between women and men coaches' perceptions regarding barriers or stigmas that women face in practice. Most women's [80% (±5%)] and approximately half of men's [54% (±5%)] believe that women coaches sometimes face discrimination and/or mistrust and most of the women's [78% (±6%)] and the majority of men's [64% (±5%)] recognize different opportunities for coaches between genders. Considering the presence of topics related to female biology in coaches' education process the majority of women [72% (±5%)] and men [63% (±5%)] considered that they were not approached enough, agreeing most of the coaches, women [92% (±3%)] and men [87% (±4%)], that coach education programmes should be more expanded to cover it. In open-ended questions coaches agreed that educational programmes can promote gender equality and mentioned a lack of contents related to equity, identity and female biology.

**Discussion:**

Finally, accepting the limitations, this survey study provides relevant contextual information and practical applications for coaching educational programmes targeting women coaches and athletes and insights for governing bodies and institutions to foster gender equality in coaching.

## 1 Introduction

Stereotypes about gender have an immediate and ongoing effect on every aspect of people's lives (Preece and Bullingham, 2022). Gendered actions are socialized and perpetuated from a young age due to the normalization of societal stereotypes (Anderson and White, 2018), enriched by traditional gender-specific roles (Spence and Helmreich, 2014). Although men and women frequently perceive these gender ideologies differently, the results are often restrictive and discriminatory (Grabrucker, 1998). Nevertheless, research can encourage future change since stereotypes are socially shaped and not absolute (Azzarito and Solmon, 2009).

Male dominance in sports is intrinsically linked to hegemonic constructions of femininity, which emphasize traits such as “delicacy” and “vulnerability” (Bell, 2008). Since the 1970s, the absolute number of women and girls participating has gradually increased, with a significant rise in participation in health and fitness programmes (Norman, 2010). Yet, these improvements in sports have not been reflected in the proportion of women in coaching and leadership positions, and the coaching profession continues to be particularly restrictive for women (Norman, 2008). Regardless of social status, ethnicity, sexual orientation, or gender identity, organized sports should be accessible to all, as sports and participation in sports promote social inclusion (Bailey, 2005) and support inclusive practices (Braumüller et al., 2020).

Gender stereotypes influence people's perceptions of themselves and others (Anderson and White, 2018), their behavior (Mulvey and Killen, 2015), their presentation (Carroll, 2019), their professional opportunities (Bird and Rhoton, 2011), and their participation in sports (Staurowsky, 2016). Women and girls are less physically active than men and boys of the same age (Women in Sport, 2022). Menstruation is one of the main reasons why young women quit playing sports or engaging in physical exercise. According to recent studies, 70% of girls avoid exercising during their menses because of pain (73%), fear of leaking (62%), tiredness (52%), and/or self-consciousness (45%) (Women in Sport, 2022). In general, female athletes from different sports and competition levels emphasize that menstruation is difficult and might have a detrimental effect on their athletic experiences (Adam et al., 2022). For young girls to benefit from physical exercise and not to consider their menstrual cycle an obstacle to participation, it is fundamental to learn how to encourage and keep them involved in sports (Keil et al., 2024).

Moreover, in a school-based context, research has shown that 42% of girls stop participating in physical education classes during their menses (Youth Sport Trust and Women in Sport, 2017). Sports participation benefits from an inclusive and diverse approach, and successful participation in sports is more likely to increase the probability of long-term commitment (Côté and Hancock, 2016). Research shows that youth sports programmes focused on play and engagement across various contexts typically enable long-term gains that align with global governments' agendas for excellence and participation (Comeau, 2013; Skille, 2011). International sports organizations and governing bodies should consider this integrative approach to provide their members with more inclusive and beneficial sports opportunities (Côté and Hancock, 2016).

Regarding coaches' knowledge, behaviors, and attitudes, it is recognized that they significantly impact youth sports experiences (Lafrenière et al., 2011), making these variables essential for the quality of coaching and the outcomes of young athletes (Li et al., 2024). Coaching entails responsibility for an athlete's physical and technical development, as well as their psychological and social wellbeing, positioning the coach as a central figure in establishing and monitoring ethical sporting behavior (Norman, 2016). In Western society, athletes' involvement in sports and their social and moral wellbeing leads to the assumption that coaches perceive their role as one that embodies an ethic of care and prioritizes ethical considerations beyond just technical aspects. In coaching, an ethic of care depends on the relationships that coaches build with their athletes, the interpretation of their knowledge and beliefs, and the effort to make coaching more inclusive and accessible (Denison, 2007).

Research shows some accomplishments in the participation of minority groups both as participants and in coaching. However, growth has been slow due to sporting organizations and governing bodies making minimal changes to their working methods, definitions, and rewards of competence. The understanding and interpretation of sociocultural issues, as well as the way in which individual and group differences are still supported, alongside the lack of changes to sports leadership, contribute to these challenges (Ely and Meyerson, 2000). As a result, persistent inequities remain in the field of sports.

Building appropriate and responsive educational tools and policies requires understanding coaches' experiences with discrimination and gathering their perspectives on potential solutions, given the critical role that coaches play in creating safe and inclusive athletic environments (Papageorgiou et al., 2023). Several educational programmes in sports have been developed to raise awareness and combat discrimination. However, it is challenging to significantly change attitudes (Dixon et al., 2016), sometimes leading to the reinforcement of generalizations or stereotypes through anti-discrimination training, which perpetuates coaches' issues when navigating the complexities of real situations (Moustakas and Kalina, 2023). A recent study stated that to combat discrimination, education must begin in schools and athletic organizations. Moreover, anti-discrimination workshops and coach education were considered essential to addressing these issues in society and as key components in fostering long-lasting inclusiveness. The same study applied a survey to investigate the content that must be included when developing a training programme for youth coaches and staff about inclusion and combating discrimination, revealing the most important specific training activities, general awareness of discrimination and diversity, and content on specific marginalized groups (Kalina and Moustakas, 2024). In this context, describing the most important content to include in educational programmes, especially targeting the needs of female coaches, remains a gap in the literature.

To support the development of the FEMCoach programme, partners conducted extensive data collection with coaches through an online survey presented and discussed in this study, aiming to analyse the needs of women youth sports coaches in practice to design coaching educational programmes that promote gender equity in youth sports.

## 2 Materials and methods

### 2.1 Participants

All participants received a freely accessible online survey invitation (via e-mail, phone, or social media) and were asked to complete the survey regarding the needs of women sports coaches. The online survey was available for data collection from July 5^th^ until October 21^st^. By the end of this period, a convenience sample of 476 practitioners from various countries and all levels of competition had completed the survey. Since this is a pilot study, *a priori* sample size calculation was not conducted. We considered the number of respondents sufficient, as indicated by previous similar studies (Ciaccioni et al., 2024; Dalamitros et al., 2023; Kalina and Moustakas, 2024). The study was approved by the Universidade de Trás-os-Montes e Alto Douro (UTAD) Ethics Committee (Ref. Doc70-CE-UTAD-2024) and was designed in accordance with the Declaration of Helsinki (World Medical Association, 2025). Participants were informed about the study's aims beforehand, and participation was voluntary.

### 2.2 Study design

A descriptive design was used for this study. The survey was developed using a web-based platform (Google Forms^®^), which facilitated data collection (https://forms.gle/RfMbJpZEEUbsbAcy5). The structure of the survey combines multiple-choice questions (i.e., only one answer allowed), checkboxes (i.e., multiple answers allowed), Likert scales, and open-ended, free-text responses to identify the perceived and actual needs of female coaches. The survey was created following a narrative review that collected scientific information on the subject. A total of 40 questions were included, divided into seven sections: (1) sociodemographic data (14 questions), (2) the inclusion environment for sports practice (three questions), (3) coach professional activities (one question), (4) menstrual cycle, hormonal contraception, and pregnancy/childcare (nine questions), (5) barriers for females in sport (five questions), (6) coaches' courses (three questions), and (7) gender differences (five questions). The survey was designed to be user-friendly, with completion taking up to 5 min. All data were collected and stored anonymously, and respondents provided informed consent.

### 2.3 Data collection method

The survey was structured into seven sections to carefully address key issues regarding women's youth sports coaches' needs in practice. This division allows us to investigate different aspects of women in sports coaching, and it was created (BR, JA) based on our group's previous narrative review, which established the current state of women's youth sports coaching education in Europe.

#### 2.3.1 Section 1: sociodemographic data

This initial section is intended to collect sociodemographic information from the participants' sample, characterizing coaches with information such as their gender, age range, nationality, race, and educational level, and contextualizing their training environments by collecting data on the countries in which the coaches currently work and where they have previously worked, alongside the duration of their coaching experience. Additionally, it considers their expertise, the age range of their athletes, the classes and genders they coach, and the number of hours they dedicate to coaching.

#### 2.3.2 Section 2: the inclusion environment for sports practice

Section 2 delves into practitioners' opinions regarding the school's environment and inclusion, gender equity and equal opportunities, and healthy practices.

#### 2.3.3 Section 3: coach professional activities

Section 3 exclusively focuses on professional activities and the degree to which coaches from different sports may have a different perspective on the hierarchisation of their professional activities.

#### 2.3.4 Section 4: menstrual cycle, hormonal contraception, and pregnancy/childcare

Section 4 aims to determine if coaches believe that there is a stigma related to the menstrual cycle, hormonal contraception, and/or pregnancy and childcare. In this section, we evaluate whether athletes use any menstrual cycle tracking software if pregnancy is a limiting factor for their careers, including these topics in the coaches' education process and coach/athlete communication regarding them.

#### 2.3.5 Section 5: barriers for females in sport

Section 5 aims to explore the cultural values associated with sports and gender, the different treatment of genders, and social justice and ethical issues.

#### 2.3.6 Section 6: coaches' courses

Section 6 approaches coaches' opinions regarding the usefulness of certain contents of learning courses, their consideration for participants' gender differences and their readiness to deal with gender identity questions.

#### 2.3.7 Section 7: gender differences

Section 7 is the final section, which evaluates coaches' opinions on gender differences and whether they believe coaches' courses can promote gender equality.

### 2.4 Data analysis

Data were extracted from the online survey (Google Forms^®^) and exported to a spreadsheet (Microsoft^®^ Excel for Mac version 16.89.1) for two types of analysis.

First, as detailed in previous research (Altarriba-Bartes et al., 2021; Pernigoni et al., 2022), absolute and relative (percentage) frequencies were calculated for categorical variables. Observed frequencies were described using qualitative terms, defined as follows: All = 100% of participants; Most = ≥75%; Majority = 55–75%; Approximately half = ~50%; Approximately a third = ~30%; Minority = <30%. Additionally, for each proportion, 90% compatibility limits were calculated (i.e., the percentage of participants that chose a particular response to a question) to evaluate the margin of error using the following formula: 90% compatibility limits = ± 1.65^*^√[*x* (100 – *x*)/*n*], where “*x*” represents the proportion and “*n*” the total sample size (Pernigoni et al., 2022).

Second, a qualitative content analysis was conducted on open-ended responses, following established methodologies (Gibbs, 2007; Krippendorff, 2004). Statements were defined as units of analysis, according to the pragmatic approach in discourse analysis (Brown and Yule, 1983). Based on previous deductive categories derived from survey sections, raw data were analysed, and thematic units and codes were inductively created to explore gender-related issues in sports coaching (Table 1). The coding process was developed and reviewed by two researchers (RF-L, VL-R).

**Table 1:** Associated categories and themes in qualitative content analysis.

**Gender-related issues associated with sports coaching**	
**Category**	**Thematic units**
Educational programmes	Topics to promote gender equality to practice sport
	Topics to promote gender equality to coach
Female-coach practice	Stereotypes and mistrust
	Leadership position for women
	Personal life
	Access criteria to coach

Categories and themes related to use in qualitative content analysis.

## 3 Results

The survey gathered a total of 476 responses, with 463 deemed suitable for further analysis. Inconsistent or incomplete data from 12 surveys led to the exclusion of those participants, and one participant was excluded due to gender categorization. Responses were categorized by gender, and it was not possible to consider “others” as a group since only one participant selected that gender option and, for this reason, was excluded from further analysis. The majority of responses came from Europe, while only a limited number originated from Africa, Asia, South America, or North America (*N* = 42).

### 3.1 Frequency analysis

#### 3.1.1 Sociodemographic data

The full participants' sociodemographic characteristics are described in the Supplementary material. Approximately half of the participants considered for analysis were women [45% (90% compatibility limits ±4%)], with approximately a third aged between 18 and 24 years old [29% (±5%)]. The other half were men [55% (±4%)], with approximately a third aged between 34 and 44 years old [27% (±5%)]. The majority of participants are White/Caucasian [82% (±3%)], and nationalities are from 24 different countries, mostly from the consortium countries: Cape Verde [6% (±2%)], Greece [19% (±3%)], Poland [8% (±2%)], Portugal [43% (±4%)], Serbia [10% (±2%)] and Spain [15% (±3%)] [93% (±2%)] and the majority of them work in these countries [91% (±2%)]. Approximately half of the female coaches have sports coaching experience of more than 7 years [44% (±6%)], and the same applies to the majority of men [56% (±5%)]. The majority of female coach individual sports [58% (±6%)], while the majority of male coach team sports [55% (±5%)]. Additionally, most of the coaches are experts in coaching [84% (±3%)], and half have either a master's degree, a PhD or a higher level of education [50% (±4%)]. Finally, the majority of female coaches [76% (±5%)] and the majority of male coaches [69% (±5%)] reported dedicating a maximum of 20 h per week to coaching. Details regarding the genders coached, the competitive level of athletes, and the age range of athletes coached are presented in Table 2.

**Table 2:** Frequency distribution of participant data characterization (*N* = 463).

**Coaches**	**Women (%)**	**Men (%)**
Total (%)	45	55
**Genders coached (%)**		
Women	94	63
Women exclusively	40	6
Men	59	93
Men exclusively	5	36
Others	5	5
**Class of athletes coached**		
World class	6	7
Elite/international level	16	23
Highly trained/national level	37	46
Trained/developmental	60	62
Recreationally active	36	36
Sedentary	7	10
**Age range of athletes coached**		
5–10 years old	46	32
11–15 years old	59	61
16–20 years old	27	39
21+	19	25

World Class, Olympic and/or World medallists; Elite/International Level, competing at the international level (individuals or team-sport athletes on a national team); Highly Trained/National Level, competing at the national level; Trained/Developmental Level, local-level representation; Recreationally Active, meet World Health Organization minimum activity guidelines (Adults aged 18–64 years old completing at least 150 to 300 min of moderate-intensity activity or 75–150 min of vigorous-intensity activity a week, plus muscle-strengthening activities two or more days a week; children and adolescents, aged 5–17 years old, doing at least 60 min per day of moderate-to-vigorous intensity activity a week, plus strengthening muscle and bone at least 3 days a week); Sedentary, do not meet minimum activity guidelines (McKay et al., 2022).

#### 3.1.2 The inclusion environment for sports practice

**Figure 1:**
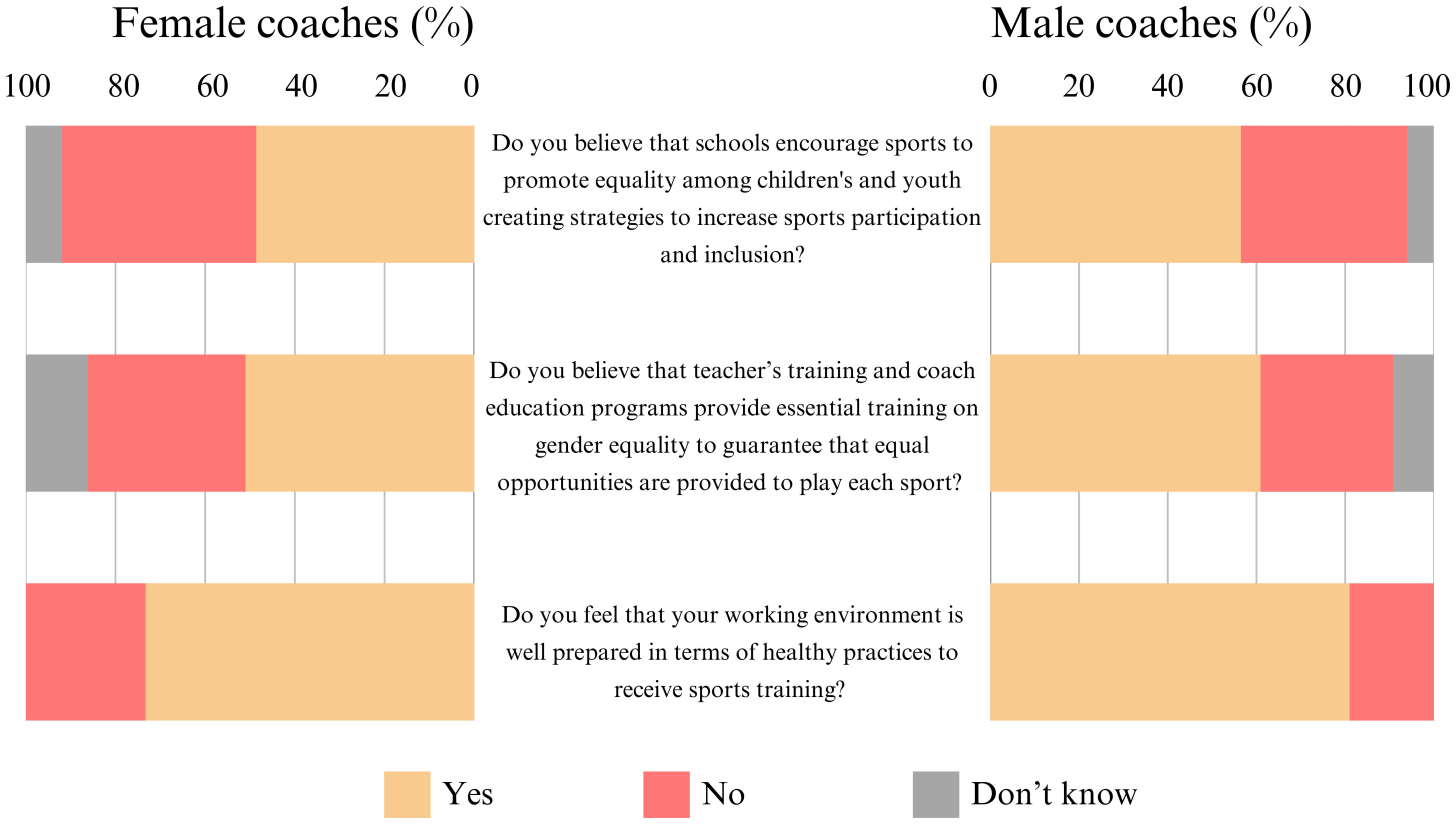
Frequency distribution of the results for Section 2: the inclusion environment for sports practice (N = 463).

Regarding the results for Section 2 of the survey (Figure 1), half of the female coaches [49% (±6%)] and the majority of the male coaches [57% (±5%)] believe that schools encourage sports to promote gender equality among children and youth by creating strategies to increase sports participation and inclusion. Moreover, half of the women [51% (±6%)] and the majority of the men [61% (±5%)] believe that teachers' training and coaching education programmes provide essential training on gender equality to ensure equal opportunities in sports. Additionally, the majority of the women [73% (±5%)] and the majority of the male coaches [81% (±4%)] feel that their working environment is well prepared regarding health practices for sports training.

#### 3.1.3 Coach professional activities

**Figure 2:**
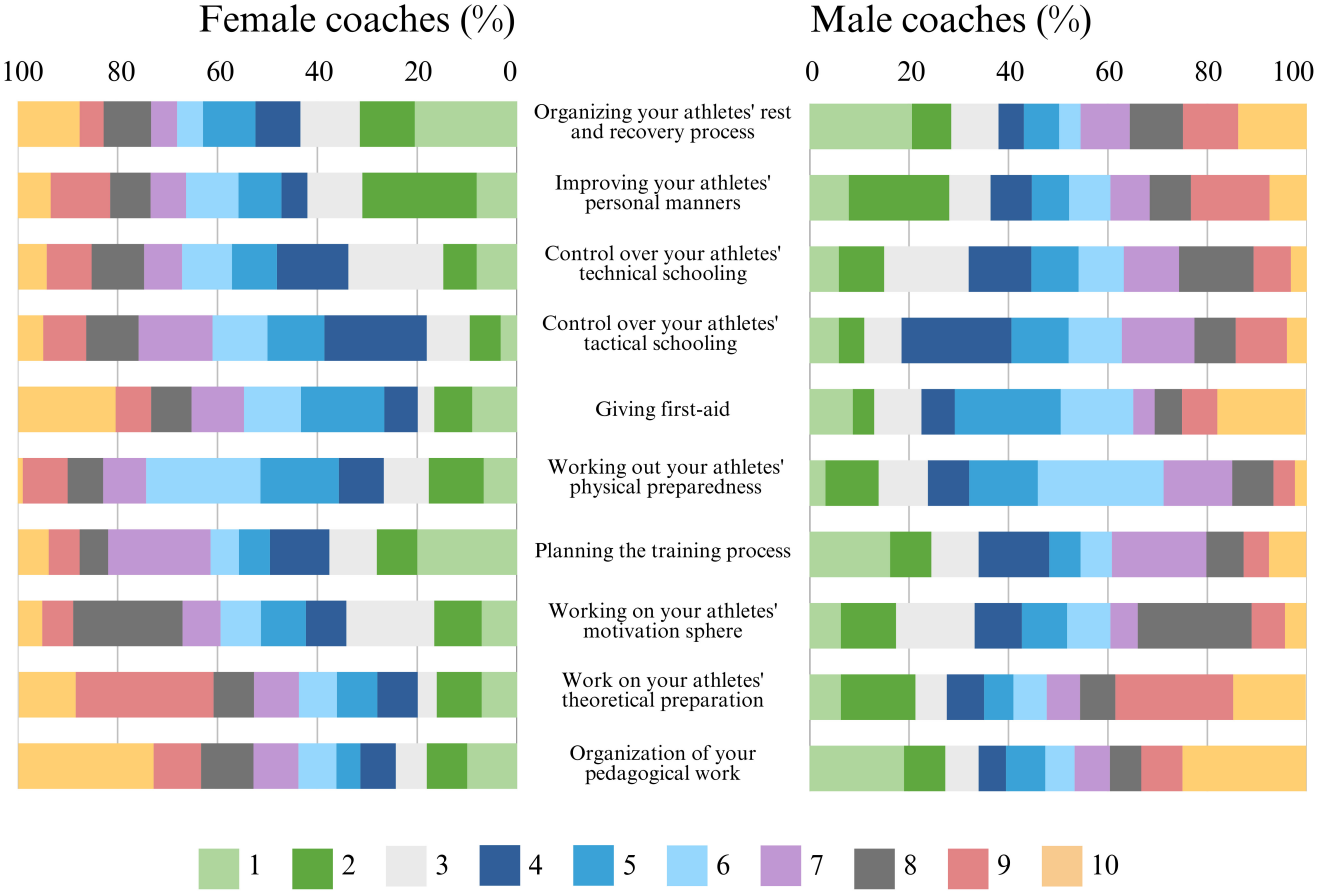
Frequency distribution of the results for Section 3: coach professional activities hierarchisation (N = 463).

Considering the results for Section 3 (Figure 2), female and male coaches shared similar opinions regarding the hierarchy of coaches' professional activities, with approximately half of all women [51% (±6%)] and the majority of men [56% (±5%)] identifying the most important activities as “organizing your athlete's rest and recovery process,” “planning the training process,” and “organizing your pedagogical work.”

In the second place, nearly half of the coaches, women [43% (±6%)] and men [46% (±5%)], similarly consider “improving your athletes' personal manners,” “working out your athletes' physical preparedness,” or “working on your athletes' theoretical preparation” for this position.

In third place, approximately half of the coaches—women [45% (±6%)] and men [40% (±5%)]—selected “control over your athletes' technical schooling,” “control over your athletes' tactical schooling,” or “working on your athletes' motivation sphere.”

Finally, in tenth place, the majority of coaches, women [59% (±6%)] and men [57% (±5%)], chose “organizing your athletes' rest and recovery process,” “giving first aid,” or “organization of your pedagogical work.”

#### 3.1.4 Menstrual cycle, hormonal contraception, and pregnancy/childcare

**Figure 3:**
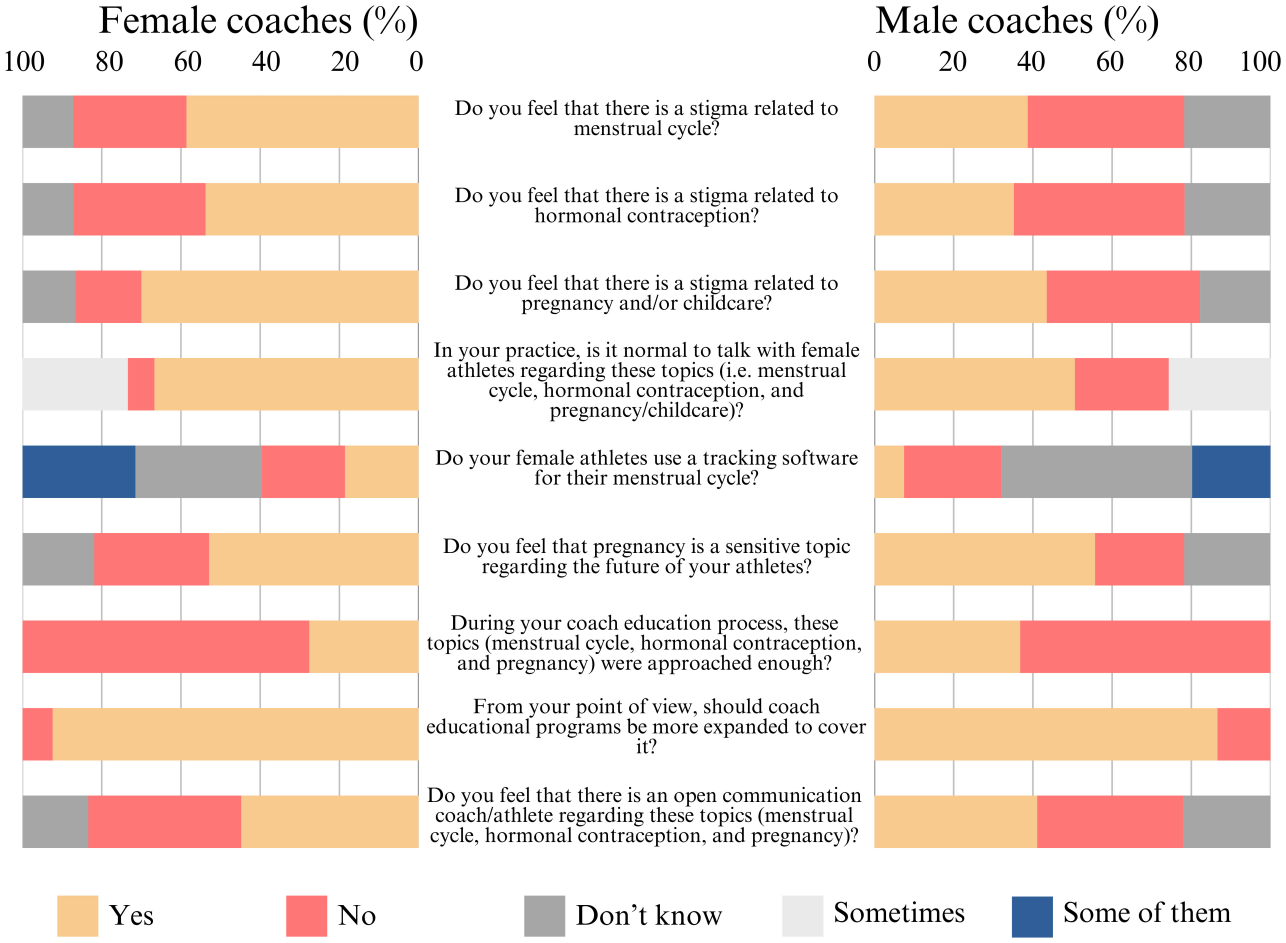
Frequency distribution of the results for Section 4: menstrual cycle, hormonal contraception and pregnancy/childcare (N = 463).

Regarding the results for Section 4 (Figure 3), concerning the topics of the menstrual cycle, hormonal contraception, and pregnancy/childcare, the majority of female coaches [59% (±6%)] feel that there is a stigma related to the menstrual cycle, while less than half of the male coaches [39% (±5%)] share this view. Half of the women [54% (±6%)] and less than half of the men [35% (±5%)] perceive a stigma related to hormonal contraception, and the majority of women [54% (±5%)] as well as less than half of the men [35% (±5%)] feel there is a stigma related to pregnancy and/or childcare. Regarding communication, the majority of women [67% (±5%)] and only half of the men [51% (±5%)] reported that discussing these topics is normal. In terms of the usage of tracking software by their female athletes, approximately one-third of women [32% (±5%)] and half of the men [48% (±5%)] indicated they do not know if their athletes use it. Additionally, half of the women [53% (±6%)] and the majority of the men [56% (±5%)] believe that pregnancy is a sensitive topic regarding the futures of their athletes. With respect to the inclusion of these three topics in the coaches' educational process, the majority of women [72% (±5%)] and men [63% (±5%)] feel they are not sufficiently addressed, agreeing with most coaches, including women [92% (±3%)] and men [87% (±4%)], that coach education programmes should be expanded to cover them more comprehensively. Finally, less than half of the women [45% (±6%)] and men [41% (±5%)] feel that there is open communication between coach and athlete regarding these topics.

#### 3.1.5 Barriers to females in sport

**Figure 4:**
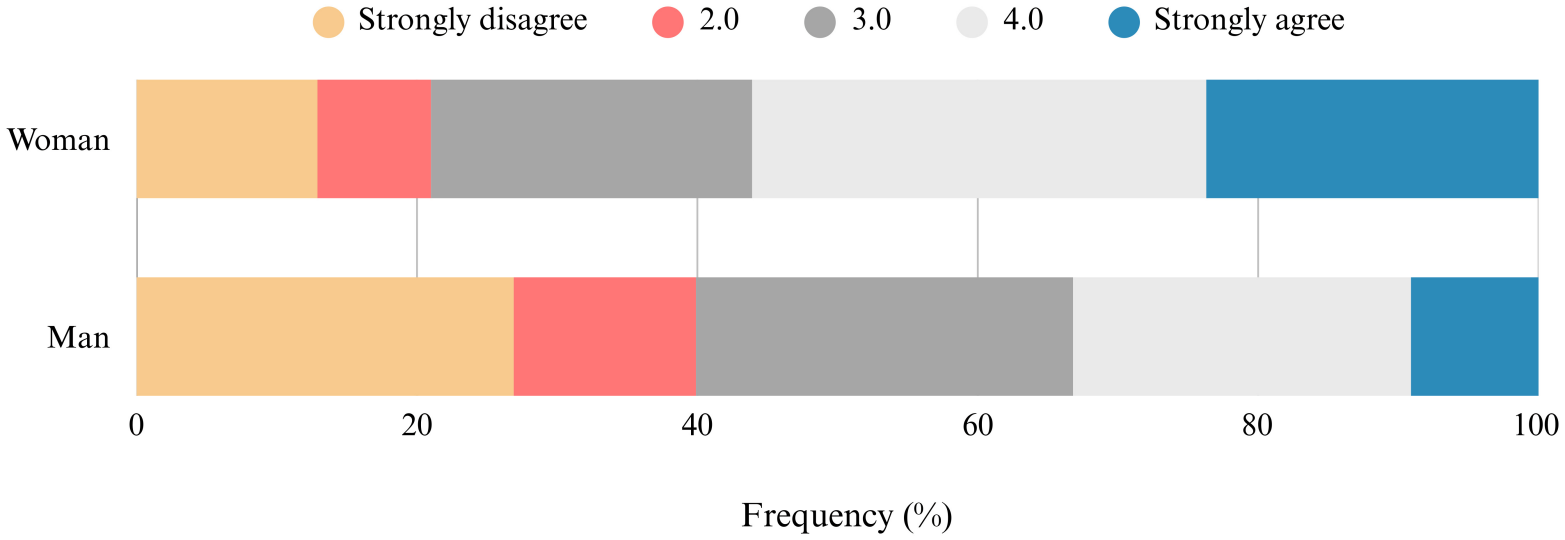
Frequency distribution of coaches' opinions regarding the following affirmation: “The cultural values associated with sport made some men believe that coaching belongs to them (N = 463).”

Concerning the results for Section 5, a minority of women [21% (±5%)] and more than a third of men [40% (±5%)] disagree or strongly disagree with the affirmation that “the cultural values associated with sport made some men believe that coaching belongs to them,” while the majority of women [56% (±6%)] and approximately a third of men [33% (±5%)] agree or strongly agree with this affirmation (Figure 4).

**Figure 5:**
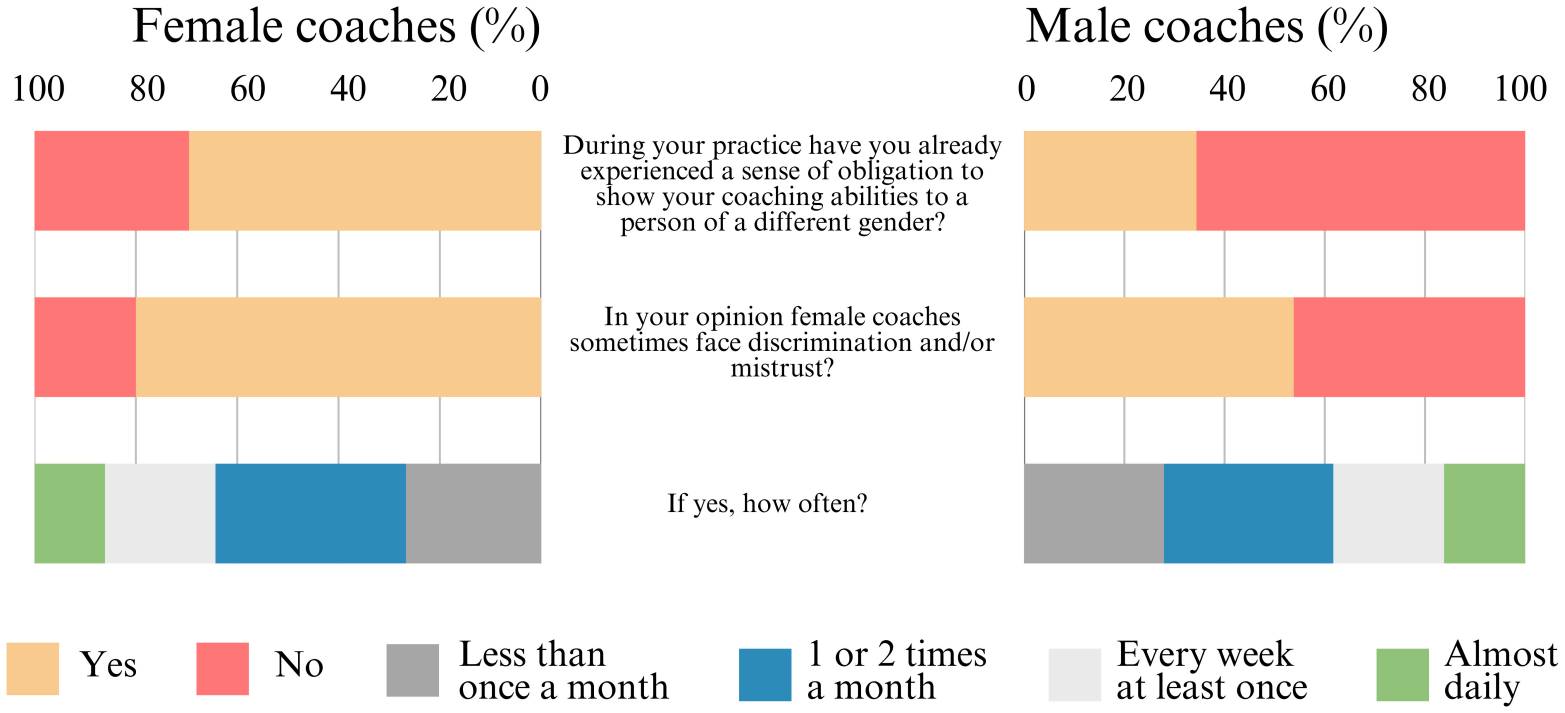
Frequency distribution of the results for questions related to barriers that women can face in sports (N = 463).

Regarding coaches' practices, if they ever felt obligated to demonstrate their coaching abilities to a person of a different gender, the majority of women responded “yes” [70% (±5%)], while the majority of men responded “no” [66% (±5%)]. A majority of women [80% (±5%)] and approximately half of the men [54% (±5%)] believe that female coaches occasionally face discrimination and/or mistrust. The frequency of this behavior is perceived by the majority of women [64% (±6%)] and men [62% (±7%)] as occurring no more than twice a month (Figure 5).

#### 3.1.6 Coaches' courses

**Figure 6:**
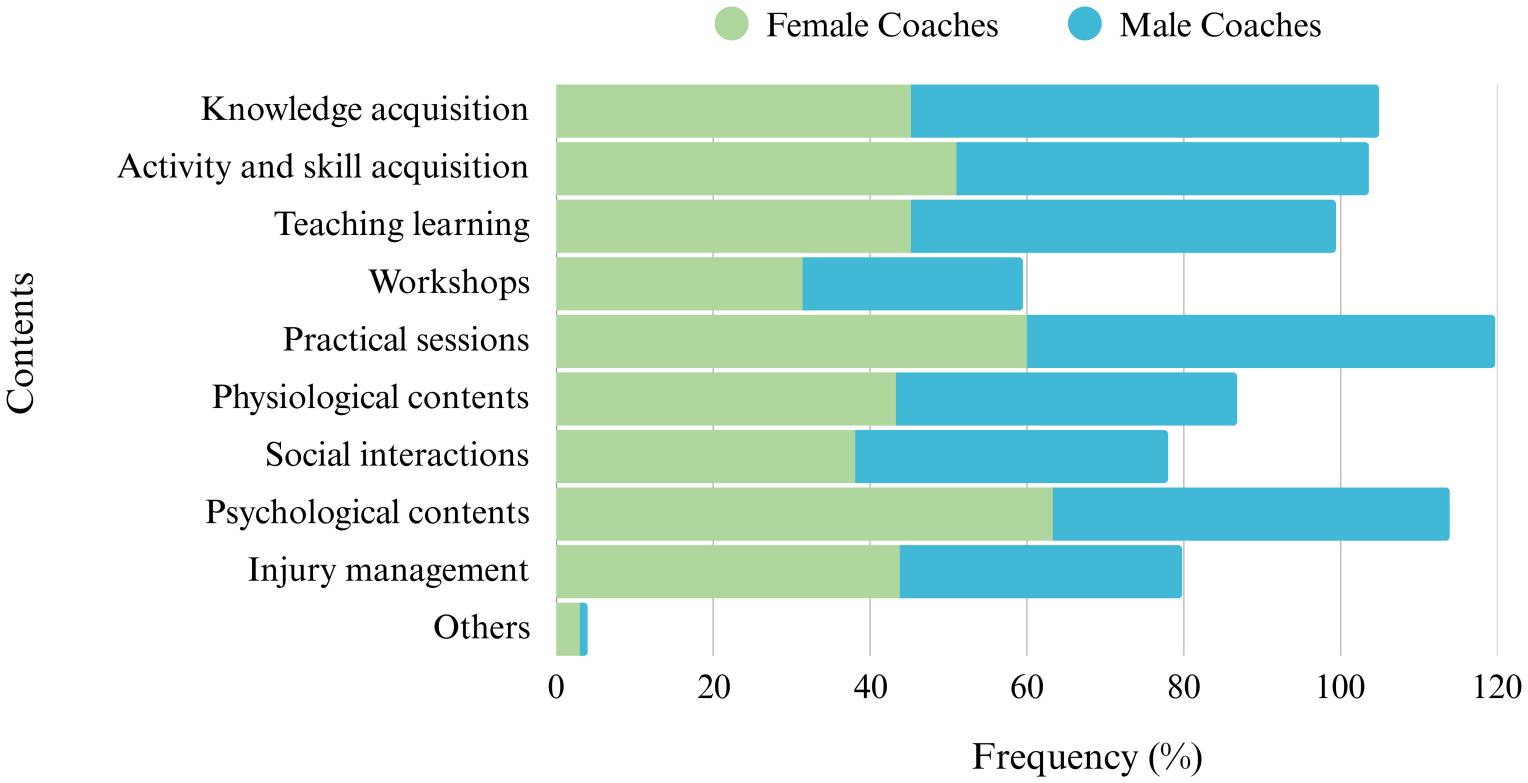
Frequency distribution of the contents of coaches' educational courses (N = 463). Others include training load, first aid, social construction of an athlete, identity, equality, menstrual cycle, mental illness, motor control, disabilities, and ethics.

Regarding the results for Section 6, the most useful types of content to include in coaching courses for women are “practical sessions” [60% (±6%)], “psychological contents” [63% (±5%)] and “activity and skill acquisition” [51% (±6%)], while for men, they are “practical sessions” [60% (±5%)], “knowledge acquisition” [59% (±5%)] and “teaching and learning” [54% (±5%)] (Figure 6).

**Figure 7:**
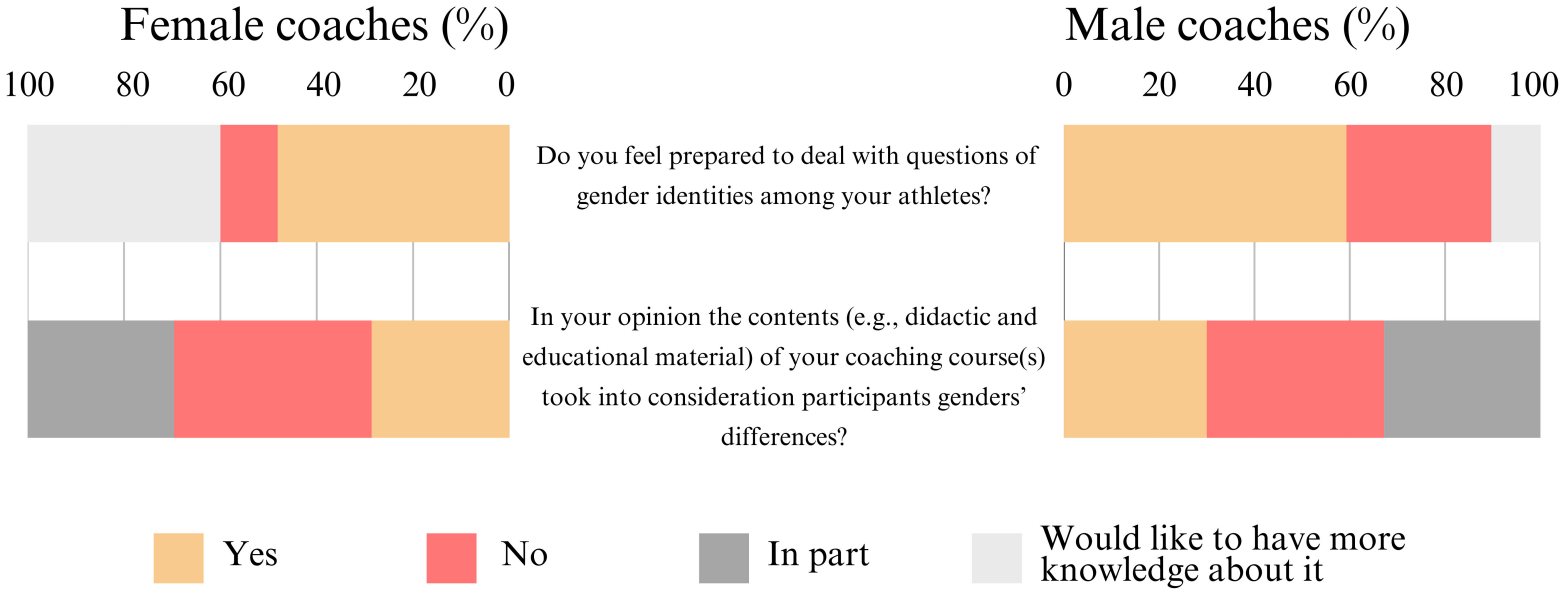
Frequency distribution of the coaches' opinions related to questions of gender identities (N = 463).

Approximately half of the women [48% (±6%)] and the majority of the men [59% (±5%)] feel prepared to address questions of gender identity among their athletes. However, over a third of the women [40% (±6%)] would like to gain more knowledge on the topic, and approximately a third of the men [30% (±5%)] reported feeling unprepared. More than a third of the coaches, both women [41% (±6%)] and men [37% (±5%)] indicated that the contents (e.g., didactic and educational materials) of their coaching course(s) did not account for participants' gender differences (Figure 7).

#### 3.1.7 Gender differences

**Figure 8:**
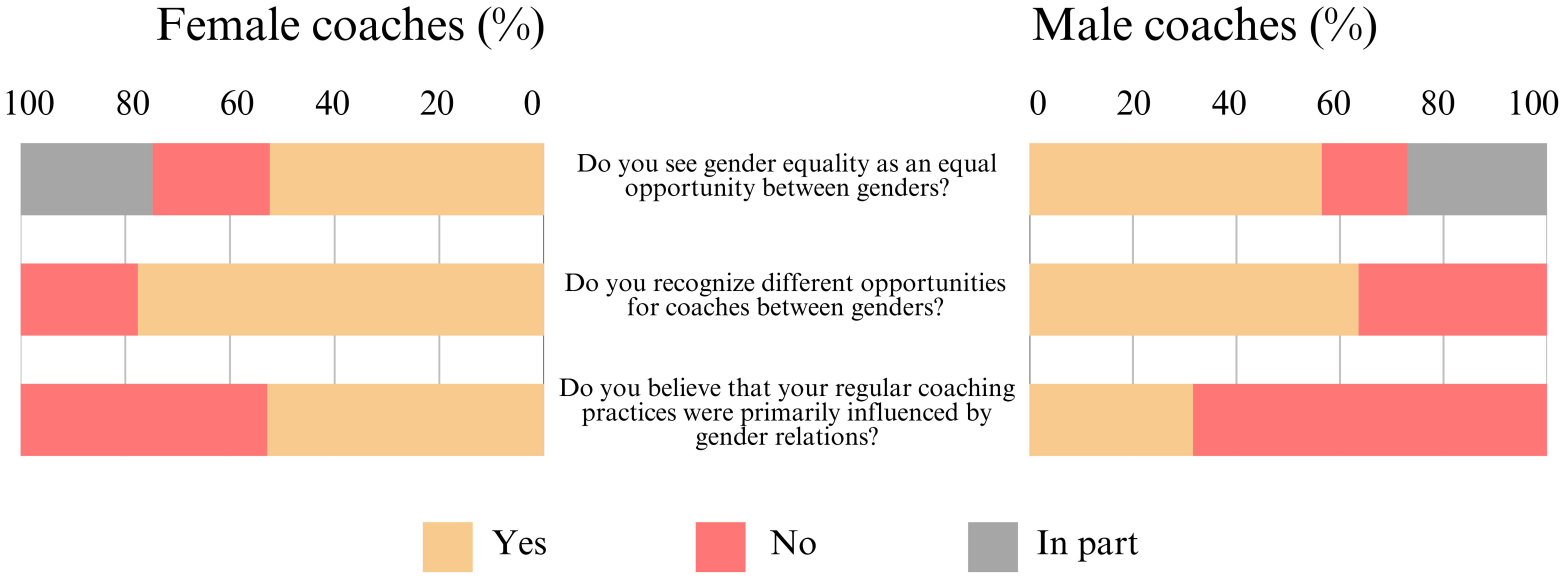
Frequency distribution of the coaches' beliefs on gender differences (N = 463).

Finally, regarding the results for Section 7 (Figure 8), approximately half of the women [52% (±6%)] and the majority of the men [57% (±5%)] view gender equality as an equal opportunity between both genders. The majority of the women [78% (±6%)] and the majority of the men [64% (±5%)] recognize differing opportunities for coaches between the genders. Approximately half of the women [53% (±6%)] believe that their standard coaching practices were primarily influenced by gender relations, while the majority of the men [68% (±5%)] did not.

### 3.2 Qualitative content analysis

#### 3.2.1 Educational programmes

Regarding coaches' opinions on the open-ended question: “In your opinion, what are the most important topics to be included in coaching education programmes regarding social justice and ethical issues?” participants often referred to the importance of *gender equality and equity in practicing sport* as topics to be included in coaches' educational programmes. Coaches' opinions frequently mentioned justice, respect, education, communication, equal rights, mental health, female biology, and empathy.

Moreover, we can read below some examples of coaches' opinions:

“Principal differences in terms of performances among gender, ages, etc., in different situations and sports.” (man, over 55 years old, Portuguese, individual sports coaches, more than 7 years of experience, male and female athletes from 11 to 20 years of age)

“Planning, programming and training specific for women.” (women, between 35 and 44 years old, Serbian, individual sports coaches, more than 7 years of experience, male and female athletes from 5 to 10 years of age)

“Awareness of the hormonal changes that your team could be experiencing according to their age. Also, how to treat and talk to them so they can feel free to talk about these topics with you.” (women between 18 and 24 years old, Spanish, team sport coach, between 2 and 5 years of experience, female athletes from 5 to 15 years of age)

“Education on the psychological, physiological, and ethical aspects of coaching transgender athletes.” (man, between 25 and 34 years old, Portuguese, team sport coaches, more than 7 years of experience, male and female athletes from 21 years of age)

Regarding the coaches' opinions on the open-ended question: “Do you believe that coaches' courses can promote gender equality?” The most frequent answer was affirmative. We can read the following opinions related to including *topics that promote gender equality in coaching*:

“By incorporating topics such as gender equality, deconstructing stereotypes, and raising awareness about the importance of diversity, these programmes can help create a more inclusive sports environment. Additionally, providing equal opportunities for access and career progression for female coaches can significantly contribute to reducing disparities and promoting a culture of equality in sports.” (man, between 45 and 54 years old, Cape Verdean, team sport coaches, more than 7 years of experience, male athletes from 21 years of age)

“By incorporating gender-sensitive training, education on bias, and strategies to foster inclusivity, these courses can help break down barriers that have traditionally limited opportunities for women in sports.” (man, between 35 and 44 years old, Greek, team sport coaches, more than 7 years of experience, male athletes from 16 to 20 years of age)

“The coach himself has to know how to deal with athletes of both genders. Everyone has the right to practise all sports. The coach has to be ready to accept and understand a girl and a boy. Regardless of the sport they practice.” (women between 25 and 34 years old, Portuguese, individual sports coaches, more than 7 years of experience, men and female athletes from 5 years of age)

“Including topics related to equality, deconstructing stereotypes, and promoting an inclusive environment can not only improve the quality of coaching but also create a more equitable sports culture. By preparing future coaches to deal with gender dynamics in an ethical and inclusive way, we ensure that sport becomes a space where all people, regardless of gender, can have equal opportunities to thrive.” (women between 45 and 54 years old, Portuguese working in Angola, team and individual sports coaches, more than 7 years of experience, men and female athletes from 11 to 15 years of age)

#### 3.2.2 Female coaching practice

Regarding the coaches' opinions related to the open-ended question: “What is your opinion regarding barriers that female coaches can face in practice.” we can read the following opinions by coaches which are related to *stereotypes and mistrust*:

“As far as gender equality is concerned, women are at a disadvantage when it comes to entering the sports labour market. There are more men as coaches in the various sports, and the macho impetus is still very much in vogue.” (man, between 35 and 44 years old, Portuguese, team sport coaches, more than 7 years of experience, male and female athletes from 5 to 20 years of age)

“Female coaches face barriers such as gender stereotypes, lack of opportunities, wage disparities, increased pressure and scrutiny, lack of representation, and challenges in balancing work and family, which limit their progress and success in sports.” (Men, from 45 to 54 years old, Cape Verdean, team sport coaches, more than 7 years of experience, male athletes from 21 years of age)

“Inequalities in perceived competence: Female coaches may be perceived as less competent than their male counterparts. Discrimination and sexism.” (woman, 25 to 34 years old, Polish, individual sports coach, between 2 to 5 years of experience, female athletes from 11 to 20 years of age)

“Gender role assumptions; leadership stereotypes, marginalisation, masculine hegemony, male-run and manage coach education, no career pathway, no merit recognition, bullying/harassment, the low payment.” (woman, 25 to 34 years old, Portuguese, individual sports coaches, more than 7 years of experience, men, women and other genders' athletes from 11 years of age)

The majority of coaches' opinions follow the same line overall. When existing, depending on the sport that we are considering and the country itself, the major barriers for female coaches are mistrust, discrimination, and the lack of parity with their male counterparts and patriarchal societies.

The still-existing stereotypes, social barriers, authority issues, prejudices, traditions and stigma related to *women in leadership positions* are also pointed out as gender differences in coaching positions. Coaches also mentioned:

“Male athletes don't respect so many female coaches.” (man, between 45 and 54 years old, Portuguese, individual sports coach, between 5 and 7 years of experience, male and female athletes from 5 to 10 years of age)

“Prejudice, questioning of competence and female leadership, professional and personal conflicts.” (woman, 25 to 34 years old, Cape Verdean, team sport coach, between 2 to 5 years of experience, female athletes from 11 to 20 years of age)

“Female coaches face lots of barriers in practice. Gender stereotypes are one of them, as many sports are viewed as male-dominated. So, female coaches struggle against the perception that men are naturally superior and suited to leadership roles. The lack of role models is another example. Female coaches are underrepresented. Young women don't have female role models to look up to.” (women between 18 and 24 years old, Spanish, team sport coach, between 2 and 5 years of experience, female athletes from 5 to 15 years of age)

There are also coaches reporting not feeling any barrier in their sport but acknowledging this issue in other sports:

“I don't notice any barriers from female coaches in the sport that I coach (…) because the sport heavily values women's empowerment. But based on what I've observed in other sports, it's quite uncommon to see women coaching men, but the opposite - that is, men coaching women - is, I must admit, fairly prevalent. It's also usual for athletes to migrate into coaching roles, and male performance in absolute terms is typically better than female performance. Therefore, I believe this is why people confuse good coaches with strong athletes and still see a male coach as being better than a female.” (man, between 25 and 34 years old, Portuguese, team sport coaches, more than 7 years of experience, male and female athletes from 21 years of age)

In relation to *personal life*, coaches reported that women sometimes struggle with:

“Pregnancy periods when related to competitive calendars, lacking some understanding from club managers.” (man, over 55 years old, Portuguese, team sport coaches, more than 7 years of experience, male and female athletes from 5 to 15 years of age)

“In my country, female coaches have the duty to raise their children and take care of the home. They cannot devote as much time as male coaches. The first years of coaching are less professionally active. When the children are older, male coaches have more coaching experience. In my opinion, this is where the difference comes from.” (women between 45 and 54 years old, Polish, team sport coaches, more than 7 years of experience, female athletes from 11 to 15 years of age)

In addition, differences in society are perceived by many coaches, who report, to some extent, positive differences between now and the past. Thus, we support a gender equality view sustained in a society that provides equal opportunities to women as it does to men, with gender just considered as a factor in coaching (as in every other aspect) because there is still a different treatment of women than there is of men just because of gender and not sustained by any other reason, and that is exactly where our concerns must be focused. All the other factors related to the professional quality of the coach must always be taken into consideration, and, from our point of view, hopefully, one day, that will be all that will really matter.

Thus, in order to promote gender equality, *access criteria to coaching* is fundamental. It is detrimental that opportunities are the same for all genders and that merit and competencies must always be the top priorities. A coach's opinion that goes in line with this is above mentioned:

“The lack of respect for a woman is, sadly, higher than for a man. Women have to face a disadvantage in order to perform their coaching skills. Although gender is now a new item in selecting a coach, (…) there's a lack of meritocracy in the process, and some women are forced to be good. There should be equal opportunities, and people should compete for places the same way, with gender independence.” (men, between 25 and 34 years old, Spanish, team sport coach, female athletes from 11 to 20 years of age, more than 7 years of experience)

“I believe that there is only one barrier, which is opportunity.” (woman, 25 to 34 years old, Cape Verdean, soccer, individual sports coach, between 2 to 5 years of experience, men and female athletes from 5 years of age)

## 4 Discussion

The main objective of this survey study was to analyse the needs of women youth sports coaches in practice to design coaching educational programmes that promote gender equity in youth sports. In this study, we explore coaches' beliefs on gender equality and identity issues and whether coach educational programmes can influence them.

The present study provides novel insights into coaches' opinions and feelings regarding gender equality and identities, ultimately providing relevant information on programmes' contents that address female coaches and female athletes, along with practical insights for governing bodies and institutions to promote gender equality in coaching.

We found that the opinions of women's and men's coaches differ regarding the barriers or stigmas that women face in practice, primarily describing feelings of mistrust and discrimination, a lack of parity compared to their male counterparts, and the influences of patriarchal societies. The majority of coaches believe that educational programmes can promote gender equality and noted a lack of content related to equity, identity, and female biology (especially the menstrual cycle and how it affects sports performance).

### 4.1 Creating stereotypes from the first contact with sports

The initial contact with sports often occurs in the early years of a child's life and is typically developed within a school context, where participants in various sports share similar physical and motor competence profiles that become more diverse as they grow older. Due to the similarities in anthropometric, physical, and motor skills profiles at a younger age, schools provide an opportunity for students to try out different sports before entering the years of specialization (Lovell et al., 2019). A more gender-divergent physical education culture that minimizes gender differences is currently more acceptable and promoted throughout Europe. Symbolic and social boundaries are gradually disappearing as a result of the social creation of a gender-neutral physical education culture and the merging of cultures (Lundvall, 2016). Positive physical education experiences enhance physical activity levels in adulthood (MacNamara et al., 2011), and understanding the facilitators and barriers to sports equips professionals with the necessary skills to promote lifelong sports and physical activity participation (Corbin et al., 2020). Negative stereotypes about gendered norms and sexuality are encountered by individuals who continue to play the sport as adults due to a lack of broader educational exposure (Grundlingh, 2010).

According to Hay and Penney (2013), societal values, cultures, opinions, political influences, and personal beliefs all play a role in the social construction of physical education. As a curricular subject, there is a distinct gendered habitus that favors particular groups in physical education (Green et al., 2007; Metcalfe, 2018), either discouraging or reinforcing specific forms of femininity and praising masculinity and conventional masculine traits such as strength, speed, and competitiveness (Anderson and White, 2018). Both teachers' and students' gendered habits and the domination of masculinity are cemented by these discourses, which are frequently reproduced and reinforced in practice (Valley and Graber, 2017; Walseth et al., 2017; Wrench and Garrett, 2017). In fact, a limited, binary conception of gender is formed by the physical education environment in schools and is perpetuated in the subject through practices, instruction, and didactic materials, making it difficult to challenge stereotypes (Anderson and White, 2018), while remaining prevalent gendered norms, stereotypical practices, and disparities (Metcalfe, 2018; Valley and Graber, 2017). The present research results reveal the need to improve teachers' training and coaching educational programmes to provide essential training on gender equality and guarantee the provision of equal opportunities in sports.

A systematic review concluded that the most frequent physical activity barriers for young girls are the lack of support from peers, family, and teachers, followed by a lack of time. The most identified facilitators for physical activity among young girls are weight loss/management and peer, family, and teacher support (Duffey et al., 2021). Physical activity promotion should attempt to implement strategies that can change the role and relationship of negative gender norms (World Health Organization, 2019). By establishing cultural expectations of what it means to be feminine or masculine (Corr et al., 2019), gender norms and stereotypes have a particular impact on physical activity participation by restricting access to and existing opportunities. Gender stereotypes are perceived by young girls as a barrier to physical activity engagement, and perceived gender dominance arises when physical activity options are viewed as socially acceptable for boys only, diminishing the value and accessibility for girls (Martins et al., 2014; Standiford, 2013). To meet the needs of young girls, curriculum development must use an inclusive approach. When creating a physical education curriculum that considers the preferences of young girls in a gender-responsive manner, an intersectoral approach, including education, sports, and health sectors, should be considered, probably requiring new policies in the field. Examples of how to adjust a curriculum for young girls include making it non-competitive and flexible, individually designed with a range of opportunities, and providing time for physical activity. The present research revealed the potential to increase schools' encouragement through sports to promote gender equality among children and youth, creating strategies to increase sports participation and inclusion. A possible strategy to increase physical activity opportunities may include policies and programmes that can promote physical activity outside of the conventional physical education setting in schools, such as active breaks and active extracurricular activities (Duffey et al., 2021). Identifying the barriers that limit participation may be simpler with more discussion of negative gender norms in the environments where physical activity occurs (Martins et al., 2014). For instance, institutions, educators, and boys should be involved in acknowledging and addressing their role in promoting these norms and developing supportive environments for girls' participation in physical exercise.

### 4.2 Female biology and sports

Regarding stigma in sports considering menstrual-related issues, research supports our findings, reaffirming the need for a change (Heather et al., 2021). Menstruating athletes face struggles due to their menstrual cycle-related physical (e.g., menstrual cramps, tiredness, bloating) and psychological symptoms (e.g., mood changes, low motivation) (Nolan et al., 2023). Athletes and coaches frequently view menstruation as a taboo, creating obstacles to open communication about the menstrual cycle (Bergström et al., 2023). Studies have shown a lack of confidence in discussing the menstrual cycle among both male and female coaches due to the delicate nature of the subject (Brown and Knight, 2022; Marais et al., 2022), limiting athletes and coaches from having timely and open discussions about menstrual symptoms and dysfunction, which is also impacted by the widespread stigma associated with the menstrual cycle and the lack of knowledge regarding this subject (Bergström et al., 2023; Findlay et al., 2020; Verhoef et al., 2021). This issue is more prevalent when male coaches are involved (Brown et al., 2021) due to a general bias and belief that the menstrual cycle is a “women's subject” (Holmes et al., 2021), which is supported by our results: fewer male coaches reported it being normal to talk about these topics. A coordinated and combined effort is needed from all stakeholders to shift perceptions of the menstrual cycle and end the social stigma associated with menstruation (Martínez-Fortuny et al., 2023).

The interaction between coaches and athletes is essential to the athletic environment, as coaches have a significant impact on players' personal and professional growth as well as their accomplishment of intrapersonal, interpersonal, and collective goals (Jowett, 2017). Athletes and coaches may promote gender equity and foster a more successful, supportive, and inclusive athletic environment if they understand their roles in managing menstrual cycle-related factors and provide appropriate assistance. Athletes frequently state that the presence of a male or female coach influences the amount and quality of communication during the menstrual cycle, indicating discomfort and insecurity when discussing the subject with male coaches (Brown et al., 2021; Findlay et al., 2020; Verhoef et al., 2021). They believe that male coaches are unable to relate to their menstrual concerns, limiting their ability to provide helpful guidance or support (Kolić et al., 2023; von Rosen et al., 2022). As a result, athletes often choose to conceal their symptoms, attributing menstruation-related pain or discomfort to non-menstrual causes (Brown et al., 2021). Studies indicate that male coaches can be reluctant to address the subject with their athletes, particularly the younger ones, while female coaches and physical education instructors are generally more open in their support and communication about the menstrual cycle, using their own experiences and knowledge of menstruation symptoms to assist in this process (Brown and Knight, 2022; Höök et al., 2021). Research also indicates that athletes tend to discuss menstrual cycles with female coaches and female staff members (von Rosen et al., 2022). Concerning male coaches, studies show their interest in learning more about how the menstrual cycle affects their athletes' training and performance, as well as how to approach and communicate with their athletes regarding menstrual symptoms (Bergström et al., 2023; Clarke et al., 2021).

To create a safe environment for young women and girls to discuss their menstrual cycle, it is crucial to de-stigmatize menstruation, enabling educators and coaches to provide young athletes with the support they need (van den Berg and Doyle-Baker, 2025). Studies conducted on female professional athletes at the senior level revealed that 76% of athletes did not talk to their coaches about their menstrual cycle despite its impact on their daily lives, training, and performance (Armour et al., 2020). Furthermore, research found that although participants believed their menstrual cycles affected their performance, only 27% of elite athletes discussed them with their coach (Solli et al., 2020). The primary reasons reported for this reluctance were shame or discomfort caused by stigma and the fact that coaches are men. Out of the 140 athletes in the study, 44% of those with female coaches discussed menstruation with their coach, whereas 22% of those with male coaches did the same (Solli et al., 2020).

Education regarding the menstrual cycle is advised for coaches and athletes. According to recent studies (Brown et al., 2021; Clarke et al., 2021), coaches receive little to no education on the subject, which is especially problematic for men as they have no firsthand experience with menstruation. Any coaching certification or course should include instruction for coaches to help destigmatise topics related to female biology (Keil et al., 2024). Education is the most crucial element in raising awareness of the menstrual cycle and encouraging open communication among athletes and coaches (Bergström et al., 2023; Clarke et al., 2021; Verhoef et al., 2021).

Between coaches and staff, it is unclear who is responsible for gathering, organizing, and sharing menstrual cycle tracking data (Marais et al., 2022). According to the results of our research, a high percentage of coaches do not know whether their female athletes are using tracking software. This may indicate a lack of knowledge regarding the benefits of this tracking for both athletes and coaches or a lack of confidence between athletes and coaches in sharing personal information.

Menstrual tracking data can be useful for understanding the menstrual cycle, identifying symptom patterns, finding coping mechanisms, customizing training programmes according to athletes' symptoms, and providing breaks when needed (Epstein et al., 2017). It is crucial to ensure that menstrual tracking data are handled appropriately due to the power dynamics between coaches and athletes, especially in elite sports, as well as to decrease athletes' dissatisfaction stemming from coaches' failure to provide follow-up information (McHaffie et al., 2022). Thus, sporting organizations should establish official procedures and structures for menstrual cycle tracking and data management, clearly defining the roles and responsibilities of all parties involved (Levy and Romo-Avilés, 2019).

In the Srinivasa Gopalan et al. (2024) review, three key factors of sporting environments that support healthy sports participation for athletes related to the menstrual cycle are recognized. (1) The presence of female coaches is significant for open communication concerning the menstrual cycle since they can relate to the struggles and experiences of menstruation (Brown and Knight, 2022). Given the historical underrepresentation of women in coaching, support, and administration roles in sports (Forsyth et al., 2019), this reinforces the need to expand opportunities and roles for women in sports leadership. (2) Exposure and experience are key as athletes learn to control and express their menstrual symptoms over time (von Rosen et al., 2022), and coaches increase their awareness (Brown and Knight, 2022). Additionally, involving older teammates in the creation of a supportive environment for younger athletes by sharing their knowledge and insights about the menstrual cycle can also help (Forsyth et al., 2023) or make athletes see their “role models” in sports talking openly about it (Keil et al., 2024). (3) In addition to gaining insight and understanding over time, athletes and coaches should emphasize comfort and trust in their relationships for open communication (Brown and Knight, 2022; Marais et al., 2022).

In line with our findings, Raudasoja and Ryba's (2024) research indicates that some individuals treat pregnancy as a taboo. Additionally, to avoid unfavorable reactions from their coaches and sports officials, pregnant athletes often delay disclosing their pregnancy (Davenport et al., 2022; Pullen et al., 2023). The participation of pregnant women in competitive and elite sports is a social issue that remains highly controversial and is viewed as a sensitive topic by many of the coaches involved in this research. Sport has historically been perceived as inappropriate for pregnant women due to its underlying logic of striving for peak performance, which often comes at the expense of other aspects of life (Douglas and Carless, 2009), thereby perpetuating a sexist culture (Fink, 2016). Davenport et al. (2023) found that coaches recognized the importance of reliable information regarding the safety of training during pregnancy. The existing guidelines are regarded as too conservative for elite athletes, and individualization, using specific benchmarks and athletes' subjective levels of discomfort as indicators, is recommended as a more effective strategy than applying a standardized schedule for all athletes participating in sports during pregnancy and returning to sports after giving birth (Davenport et al., 2023). In both traditional and social media, pregnant athletes are often portrayed as irresponsible, with sexism, particularly sexual objectification and unfair discrimination, identified as the primary factors contributing to this stigmatization (Weaving, 2020).

Considering the presence of these topics related to female biology in the coaches' education process, the results of the present research reinforce that they are not sufficiently addressed and that coach education programmes should be expanded to cover them. It is recommended that coaches frequently update their knowledge on issues related to female biology and maintain a clear and understandable manner of speech for athletes' comprehension. As our results confirm the lack of open communication between coaches and athletes regarding these topics, changes must be implemented. Additionally, since gender education is a powerful tool for reaching all age groups and influencing broader social change, it is recommended that mandatory content be created in schools (Raudasoja and Ryba, 2024).

### 4.3 Gender as a barrier to sports coaching

Overt or structural causes are not always the reasons for the barriers that prevent women from achieving positions of power in sports. Conversely, relatively minor events often shape women's experiences as coaches. Some men believe that coaching is primarily a male domain due to the cultural values associated with sports, and women who aspire to be successful often encounter feelings of doubt and mistrust from their peers. Consequently, female coaches often feel pressured to demonstrate their coaching abilities to men (Norman, 2010), which is supported by the present research results, where the majority of women indicated that they had experienced a sense of obligation to show their coaching skills to someone of a different gender, while most men reported that they have never experienced this. When asked if they agreed with the assertion: “the cultural values associated with sport made some men believe that coaching belongs to them,” coaches' opinions varied significantly, with the majority of women and only a third of men agreeing with this statement, reflecting differing understandings between genders. Moreover, male and female coaches' opinions diverge when asked about their beliefs regarding whether female coaches sometimes face discrimination and/or mistrust; in this case, the majority of women, but only half of the men, agreed with the statement.

According to Aitchison et al. (1999), the industry where women face the greatest discrimination and harassment in sports. Everhart and Chelladurai (1998) studied gender disparities in preferences for a coaching career and examined discrimination as a potential barrier for women pursuing coaching. Female coaches encounter numerous obstacles due to the unbalanced organizational structures prevalent in the coaching industry. Research in this field has demonstrated that women often receive less for their efforts than men, yet they quantitatively express some degree of satisfaction working within these institutions. Three retention factors (i.e., recognition and collegial support, inclusion, and working conditions) have been identified as significant in shaping work experiences. Despite women stating that inclusivity is an important aspect of their work experience, more men reported feelings of inclusivity (Pastore et al., 1996). In the present research, differences can be found in how men and female coaches perceive these ideas, reinforcing the need for greater awareness and the development of clear actions to combat the real barriers that women still face in their coaching careers.

Coaches must develop the ability to identify and address social justice concerns by becoming aware of social disparities, the unequal distribution of power, and ethics in both their actions and those of others. This process can also encourage coaches to engage in deeper self-reflection (Cushion et al., 2003). According to Culp (2014), 43 student coaches from the United States participated in a 16-week social justice course based on social constructivism. Throughout the course, topics related to inequality were introduced and explored. Analysis after the course revealed shifts in coaches' social justice ideologies, a greater understanding of under-represented groups' perspectives, and an enhanced critical perspective when analysing sports procedures. In the same study, results suggested that coaching educational programmes might benefit from emphasizing constructivism and democratic education (Culp, 2014).

The findings of Norman (2016) showed that viewing gender equity through an “equal opportunities” lens leads to a narrow conceptualization of such issues by coaches. This perspective fails to challenge dominant and discriminatory ideologies and does not enable coaches to effectively address equity within their practices. Consequently, coaches struggle to understand the importance of managing these issues. While equal opportunities between genders in sports coaching are a necessary step, they are certainly not sufficient to address and solve this issue when considering the bigger picture.

Equality and equity are multifaceted ideas that encompass distribution among groups, access to resources, the quality of interpersonal relationships, and whether people relate as equals (White, 2007). When discussing equality, Baker et al. (2009) raised several important questions, such as: (1) to understand equality, it is necessary to recognize the patterns of inequality, (2) these patterns need to be challenged using a suitable framework, (3) the goals of equality must also be determined, and (4) the best institutional structures for attaining equality in various situations must be established. This should outline the institutional strategies that would be most effective in fostering equality within a given setting.

To ensure equity (i.e., to mitigate potential inequities for people and groups), various institutions, sectors, and organizations work toward equality (i.e., everyone has equal opportunities and access to the system) through various projects, programmes, and services.

Sports continue to be considered as an inequitable, gendered, hegemonically male institution. Thus, identities, organizational practices, and processes of control and action are differentiated between men and women largely dominated by men (Fielding-Lloyd and Meân, 2011). In the present research, most coaches recognize different opportunities for coaches of various genders. Several coaches view gender equality as an equal opportunity between genders, and working to enhance inclusion through an equal opportunities ideology has the advantages of challenging inequality in sports, eliminating unfairness, and increasing the likelihood of minority groups being employed, trained, and retained in leadership roles (Ely and Meyerson, 2000). However, instead of scrutinizing organizational structures and cultures, an “equal opportunities” approach shifts the focus to external variables as the reason for under-representation (Lusted, 2009; Shaw and Slack, 2002).

Regarding gender relations, previous research has emphasized that they are at the heart of women's sporting experiences and constitute motives for their under-representation in sports leadership or for unequal athletic experiences (Norman, 2010). In Norman's (2016) study, participants' experiences revealed that gender relations, intersecting principally with religion and ethnicity, supported their everyday coaching practices. In the present research, considerable differences between men and women were observed when asked if their regular coaching practices were primarily influenced by gender relations, with a greater impact reported by women.

Instead of being understood as power dynamics or factors influencing how people perceive their lives, aspects of an individual's identity are viewed as “add-ons” (Ely and Meyerson, 2000), contributing to a narrow and weak approach to these questions among coaches and athletes (Norman, 2016).

### 4.4 Coaches' education for a paradigm change

Independent of gender, the differences in the hierarchisation of coaches' professional activities and the type of sport coached are well established. Thus, the perception of coaches changes depending on the sports' needs (Sterkowicz et al., 2007). Over the years, professional activities have been described, developed, and studied across various sports disciplines (Sterkowicz-Przybycień and Purenović-Ivanović, 2021). A hierarchy of their importance related to sports success has emerged from previous publications that considered coaches' perspectives on the value of professional activities in different sports [e.g., judo (Sterkowicz et al., 2007), artistic gymnastics (Sterkowicz-Przybycień and Purenović-Ivanović, 2021), taekwondo (Bujak et al., 2012), and karate (Dobrzycki, 2020)]. This hierarchy can be applied to enhance the educational programmes for upcoming generations of coaches in these fields (Sterkowicz-Przybycień and Purenović-Ivanović, 2021). The results of the present research indicate that coaches' gender did not have a considerable impact on the hierarchisation of coaches' professional activities. However, more research should investigate whether there are differences in coaches' perceptions of the hierarchisation of professional activities, considering specific sports and the gender of the coach. Researchers acknowledge the need for further investigation in this area to enhance current coaching educational programmes (Radojević et al., 2019).

When studying the individual impact of coaches' educational programmes, the research mentioned the need for an adaptive approach, considering genders (e.g., girls/women may benefit more from content related to physical activity and self-esteem than boys/men) (Douglas Coatsworth and Conroy, 2006; Guagliano et al., 2015). The present research results reinforce the need for a gender-sensitive approach when creating coaching educational programmes, considering the different degrees of usefulness that coaches attribute to various types of content to be included in such programmes. In addition, according to the results, they could also benefit from a reformulation of the didactic and educational material, considering participants' gender differences. This also emphasizes the importance of future studies to create large-scale interventions designed specifically for girls, particularly when female coaches supervise them. The complex effects of coaching highlight the need for a comprehensive approach to coaching education that incorporates the numerous demands and experiences of athletes and the progress of coaches (Li et al., 2024).

Over the past 10–15 years, the effectiveness of coaching for youth has begun to include more robust outcomes, such as identity, mental health, social-emotional wellness, and moral education, as well as outcomes focused on equity and justice (Newman et al., 2024; Zeisner and Smith, 2022). It is important to understand that several variables appear to affect coaching effectiveness, including team individuality and coach dynamics, athletes' age and gender, the type of sport, how coaching educational programme interventions are conducted, and the backgrounds of coaches (Li et al., 2024).

It became clear from the results of the present research that not all coaches feel prepared to address questions of gender identity among their athletes and that they wish to gain more knowledge about this matter. This also highlights the need to incorporate this topic into coaching educational programmes to promote inclusivity. Everyone involved in sports (i.e., coaches, educators, athletes, administrators, doctors, and so on) should receive training on how to prevent heteronormativity from being reinforced and how to foster supportive environments, equipping them to prevent, identify, and address prejudice and discrimination due to their crucial role in translating policy to the field (Kavoura and Kokkonen, 2021).

A scoping review found that addressing some problems faced by athletes and coaches, including gender and sexual minorities in sports, could be improved by combining the implementation of anti-discrimination policies with gender and sexual diversity education for all involved (Kavoura and Kokkonen, 2021). Existing discriminatory ideas about gender and sexuality continue to thrive at a micro level due to the lack of strategic direction, actions, or guidance from national governing bodies, as well as inadequate sociocultural education for coaches (Norman, 2013). By highlighting the importance of inclusivity and exemplifying alternative approaches to institutional structures, athletic departments can act as “social change agents” and influence the sports environment for transgender athletes as well (Cunningham, 2015). There will likely be more educational opportunities through continuing education programmes and professional conferences as research on transgender needs and experiences progresses (Munson and Ensign, 2021).

## 5 Conclusion

To the best of the authors' knowledge, this study is the first to analyse the needs of women youth sports coaches through their opinions on the educational programmes' content and the perceived barriers and stigmas related to female coaches. The majority of participants recognized gender differences in the sports coaching profession and agreed that coaching programmes represent a primary approach to a paradigm shift. Therefore, it appears that theoretical and practical aspects align with the evidence of perceived needs among female coaches, which may serve as a starting point to help optimize both female coaches' careers and the development of female athletes by adapting coaching training to female biology.

Starting from what is, for many, the first contact with sports in physical education, schools must encourage sports to promote equality among children and youth, creating strategies to increase sports participation and inclusion. It is important to ensure that teachers' training and coaching educational programmes provide essential training on gender equality to guarantee equal opportunities to play each sport.

Regarding the menstrual cycle, hormonal contraception, and pregnancy/childcare, a significant stigma is associated with these topics. Their relevance for open communication in coaches' educational programmes may stand as a first step to start changing mentalities and help coaches deal with these topics among female athletes, ultimately improving their performance. A discussion on gender identities must also be included as part of coaches' education so they can easily comprehend and manage issues that athletes may face. Concerning barriers for women in sports, the cultural values associated with sports have helped create some of them, and clearly, the sense of mistrust or discredit is still strongly present in the population of female coaches.

Regarding coaches' professional activities, the results indicated a similar pattern across genders. However, further research should explore whether different sports and/or the gender of the coach will influence these results, which must be considered in coach education. Finally, coaching educational programmes should adopt a gender-sensitive approach and consider coaches' gender adaptation of the content to their specific needs. These actions will help reduce gender inequalities and contribute to promoting equal opportunities between genders.

Considering the results obtained and acknowledging the limitations, we can conclude that this survey study provides relevant contextual information and practical applications that may be useful for educational programmes aimed at women sports coaches and female athletes. Additionally, it offers insights to promote gender equality and inclusivity in coaching, aspiring for equal opportunities between the genders in the coaching profession.

## 6 Strengths

The main strength of the present study is that it provides a comprehensive approach and an up-to-date panorama of coaches' perceptions regarding the existence of gender differences in the coaching profession, considering both women's and men's opinions on the sports coaching profession from diverse backgrounds and countries.

This study represents an innovative investigation into female coaches' needs and barriers within their profession, opening the discussion about gender equity in coaching. By acknowledging gender differences in coaching, essential insights are provided regarding the challenges that female coaches face in practice, creating an important approach to develop the necessary strategies to improve their opportunities and, consequently, their professional careers. By reporting the stigmas related to female biology and highlighting the importance of addressing these topics in coach education programmes, we foster a supportive environment for female coaches and athletes. The discussion on the significance of specific topics in coach educational programmes encompasses various fields and reflects the current and actual needs of female coaches to implement conscious changes and raise awareness about the need for shifting mentalities.

In summary, the study's strengths lie in its innovative focus, new insights, and significant contributions to the discussion concerning gender equity within the coaching profession.

## 7 Limitations

The present study acknowledges some limitations that should be considered, primarily related to the data collection instrument. The use of online surveys may result in varied interpretations of the questions posed, depending on the respondent. Although efforts were made to employ simple and precise language while keeping the questions concise and offering multiple options, along with extensive testing of the survey prior to distribution, misinterpretations may still have occurred. To reduce the impact of incorrect assumptions, we provided the lead researchers with contacts in case coaches required clarification. Additionally, the survey did not undergo a validation process for internal consistency or reliability of the collected responses (e.g., test-retest reliability), which may introduce some ambiguity into the final data.

Another potential limitation is the possibility of a response bias, as the largest proportion of survey answers were from Portugal. Thus, possible stereotypes and cultural differences among participants may have affected the results (Kemmelmeier, 2016). Moreover, it is necessary to recognize the different degrees of emphasis given by each country to sports, in general and in specific types of sports. Moreover, a potential strategy to mitigate cultural bias should be considered for future research, including authors from different nationalities to analyse the results.

Furthermore, potential biases emerged from the convenience sampling approach since the survey was designed to be openly accessible to coaches and was advertised across various platforms (e.g., email, phone, social media), allowing all interested coaches to participate. This enrolment strategy limited the authors' ability to obtain crucial information regarding the practitioners who participated in the survey and those who did not (e.g., the rate of participation or rejection in each group).

Moreover, the results of the presented survey must be interpreted with caution because (1) the a priori sample size calculation was not performed, and although we considered the number of responders to be adequate in relation to the sample size of previous similar studies (Ciaccioni et al., 2024; Dalamitros et al., 2023; Kalina and Moustakas, 2024), the compatibility limits of proportions appear, in some cases, inadequate for comparison, suggesting that a larger sample size would be required, and (2) the uncertainties for comparisons of proportions are, in some cases, too large to draw confident conclusions.

Finally, the survey was developed exclusively in English, possibly making some coaches unable to participate, which created a sample bias regarding the English-speaking practitioners willing to participate in the study.

## 8 Practical applications

Despite the study's limitations, the results from this research may be applied in the development of sports coach educational programmes, as they contribute to a deeper understanding of coaches' perceptions of gender equity among peers, emphasizing key issues and areas for intervention. These educational programmes would specifically address women's needs, helping coaches engage with their athletes and confront the stigma associated with topics such as the menstrual cycle, hormonal contraception, and pregnancy/childcare. The educational programmes can also include structured discussions concerning gender identity and cultural perceptions of gender in sports.

The present study may also encourage reflection among schools, governing bodies, and institutions regarding inclusivity and gender equity in educational programmes, raising awareness of gender biases. Eventually, this could lead to the development of strategies to increase the participation rates of women and girls in sports and physical activities, as well as to ensure equal opportunities for all children and youth.

**Figure 9:**
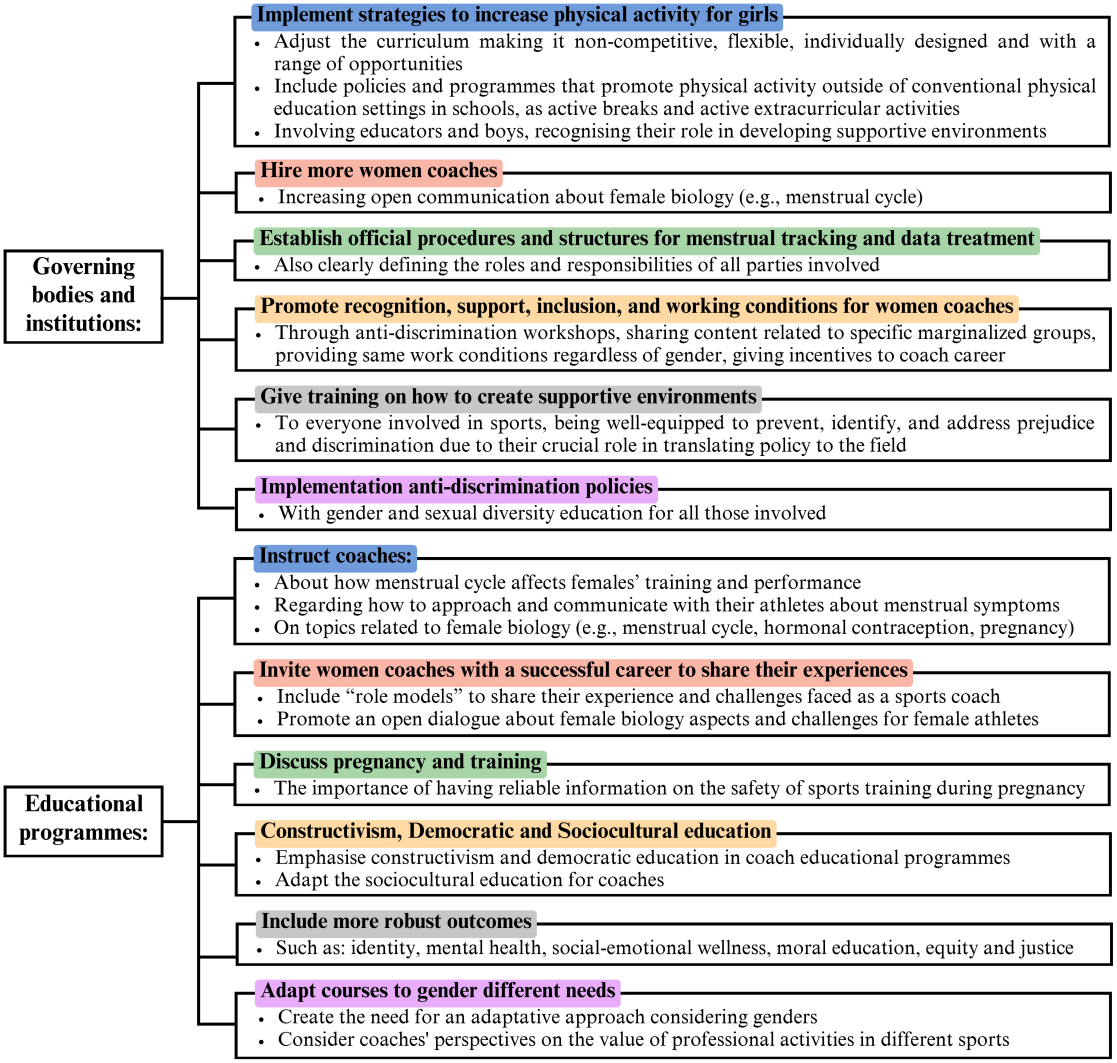
Key findings to promote gender equality in sports coaching.

In conclusion, the practical applications of this study emphasize the importance of targeted educational programmes and awareness movements that directly address the needs of female coaches and contribute to a more equitable coaching landscape. This strategy may lead to meaningful improvements in the coaching profession and foster a more inclusive environment for all athletes (Figure 9).

## 9 Future lines

The present study establishes a foundation for ongoing research efforts by identifying several areas for further investigation. Longitudinal studies could assess the effectiveness of the proposed interventions and the long-term effects of adapting sports coach educational programmes to a more gender-sensitive approach.

To strongly support and solidify the results on this subject, future research should recruit more practitioners to resolve the sample size problem, develop a more effective recruitment method to minimize potential sampling bias and explore new directions in cross-cultural differences.

Following these future lines, we will be able to enhance the foundational knowledge gained from this study, ultimately striving for significant progress in gender parity within the sports coaching field in Europe.

## Data Availability

The raw data supporting the conclusions of this article will be made available by the authors, without undue reservation.
