# Supplementary material for: Analysing gender disparities in youth sports coaching: an international survey (FEMCoach project)
Source: Front Psychol. 2025 May 7;16:1560764. doi: 10.3389/fpsyg.2025.1560764 (PMC12092410; doi:10.3389/fpsyg.2025.1560764)
Supplement: Supplementary file 1 [file Supplementary_file_1.docx]

Supplementary Material


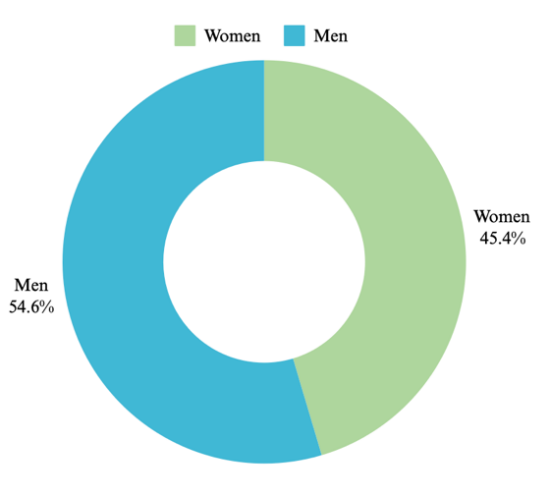


Figure 1 – Frequency distribution of survey responses considered for analysis.

Sociodemographic Data


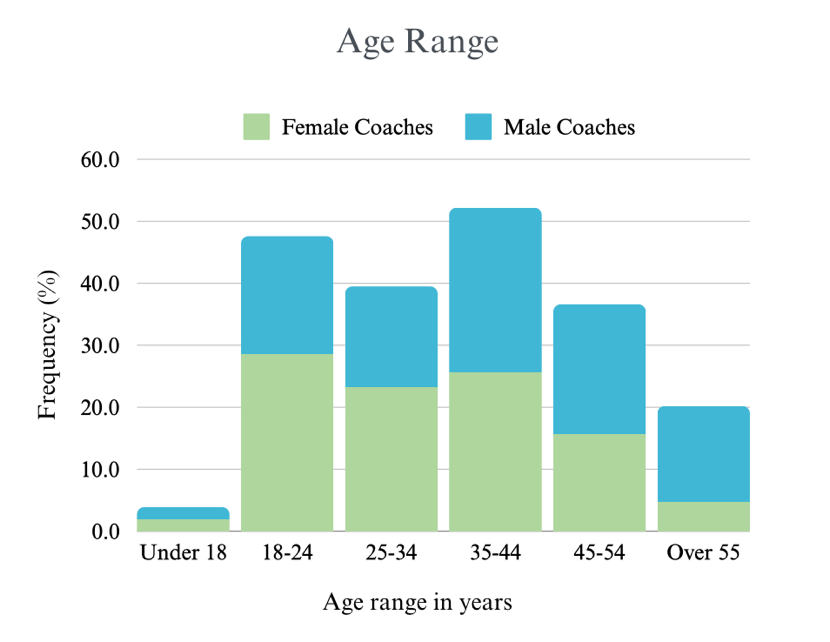


Figure 2 – Frequency distribution of the age range of coaches (N = 463).


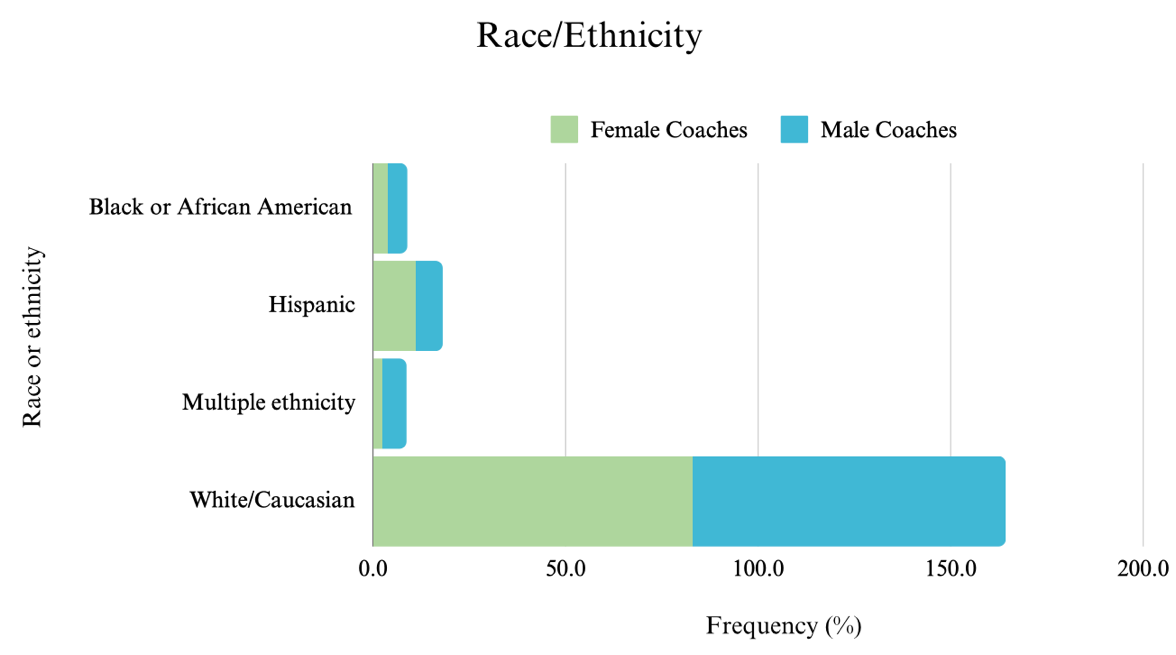


Figure 3 – Frequency distribution of the coaches’ race or etnicity (N = 463).


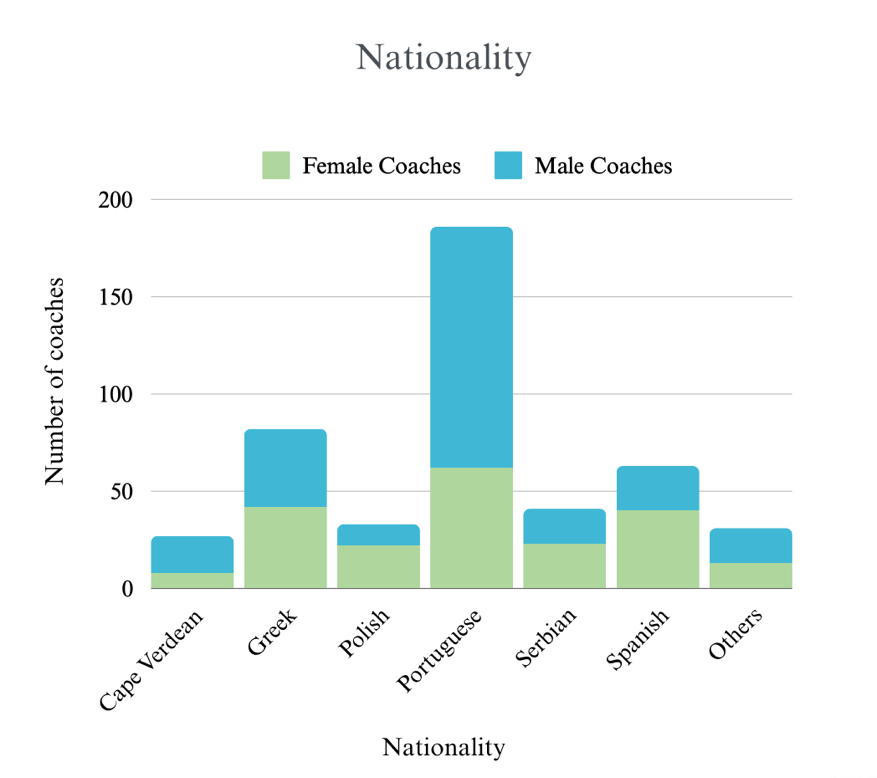


Figure 4 – Nationality of coaches (N = 463). Notes: Others include American; Angolan; Albanian; Brazilian; Costa Rican; Chilean; Croatian; Cypriot; German; Hungarian; Italian; Kenyan; Lithuanian; Macedonian; Mexican; Montenegrin; Ukrainian.


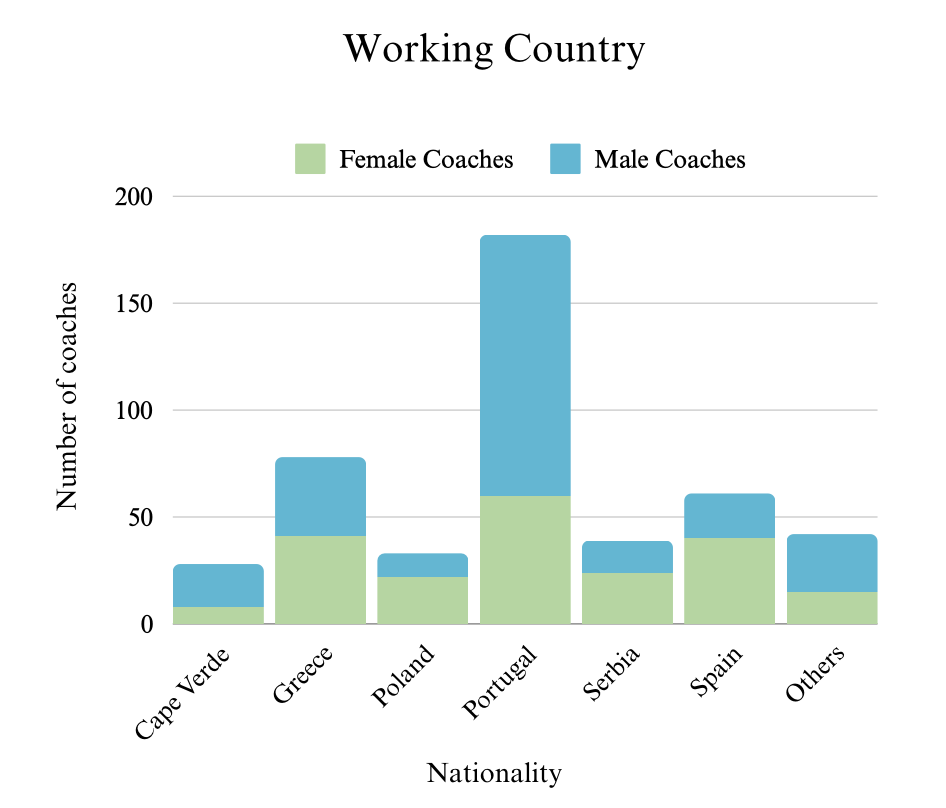


Figure 5 - Nationality of coaches (N = 463). Notes: Others include: Angola; Bosnia and Herzegovina; Brazil; Canada; China; Croatia; Costa Rica; Cyprus; Germany; Hungary; Italy; Kenya; Kosovo; Lithuania; North Macedonia; Mexico; Sweden; Switzerland; United Arab Emirates; USA


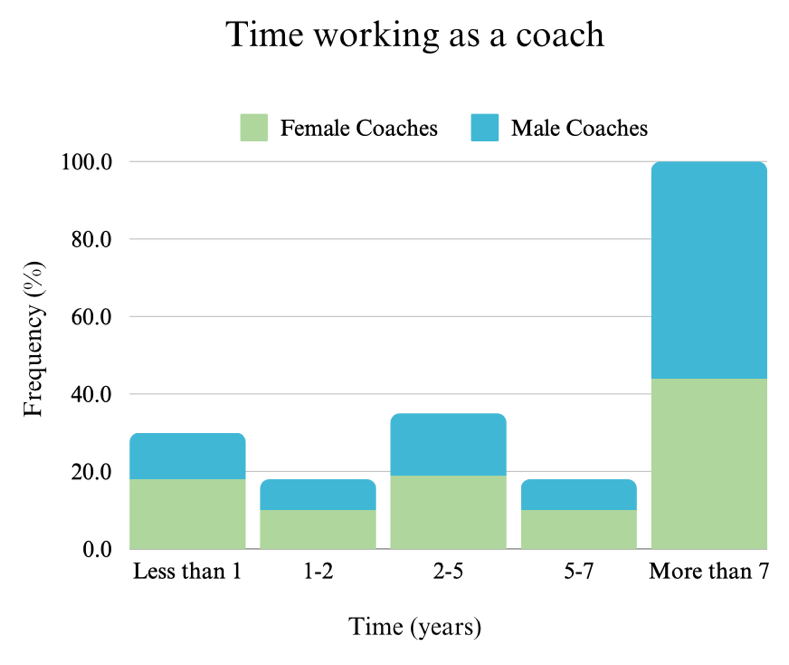


Figure 6 – Frequency distribution of the experience in coaching (N = 463).


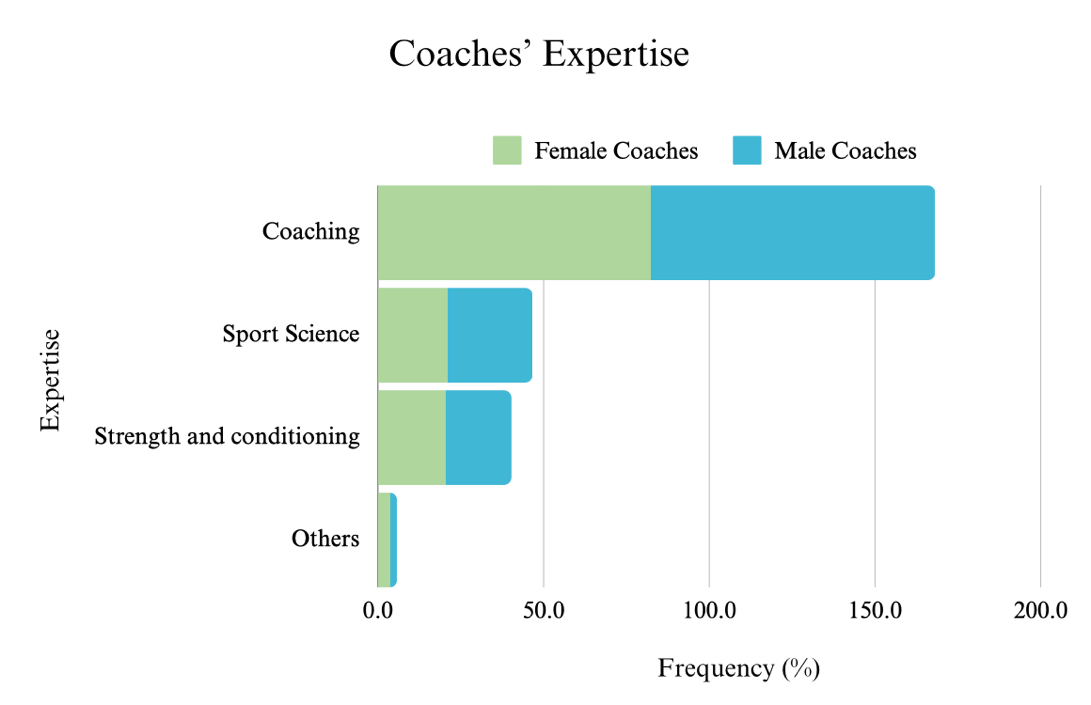


Figure 7 - Frequency distribution of coaches’ expertise (N = 463). Notes: Others include: Athlete; Physical activity and health; Physical education; Pilates; Psychology; Sports Director; Researcher; Sports and disabilities; Data analyst.


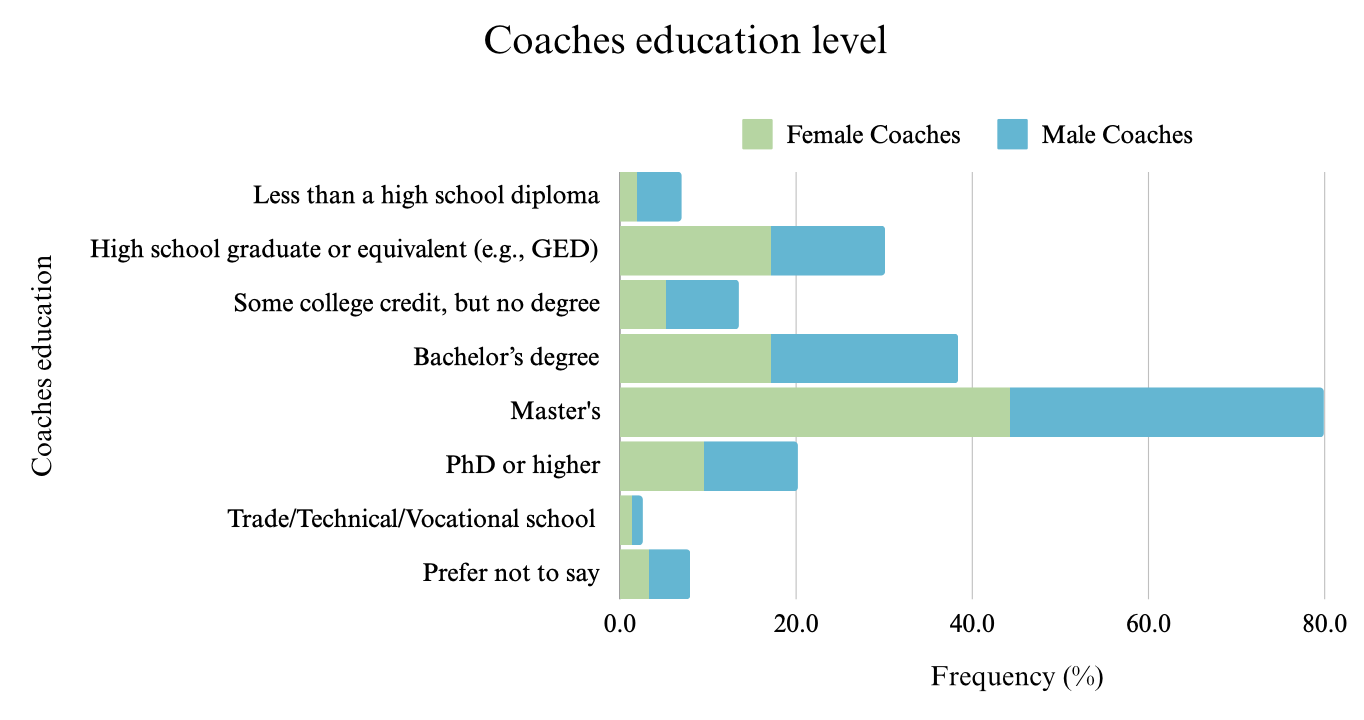


Figure 8 - Frequency distribution of the of the highest degree or school level that coaches have completed (N = 463).


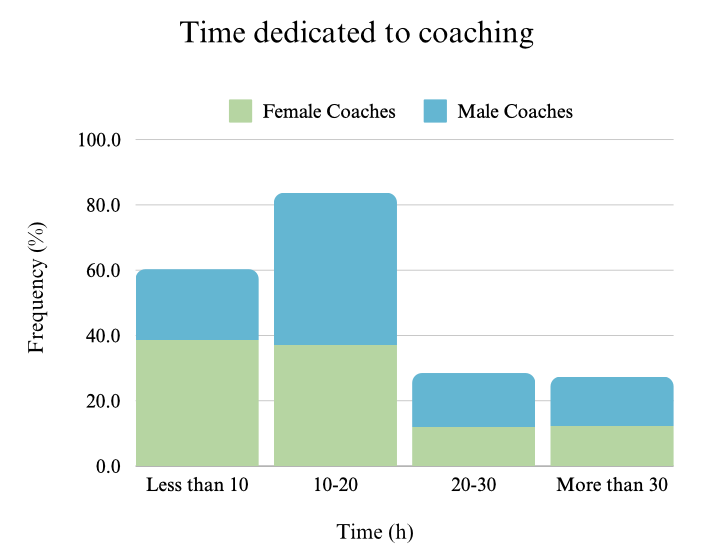


Figure 9 - Frequency distribution of the time dedicated to coaching per week (N = 463).


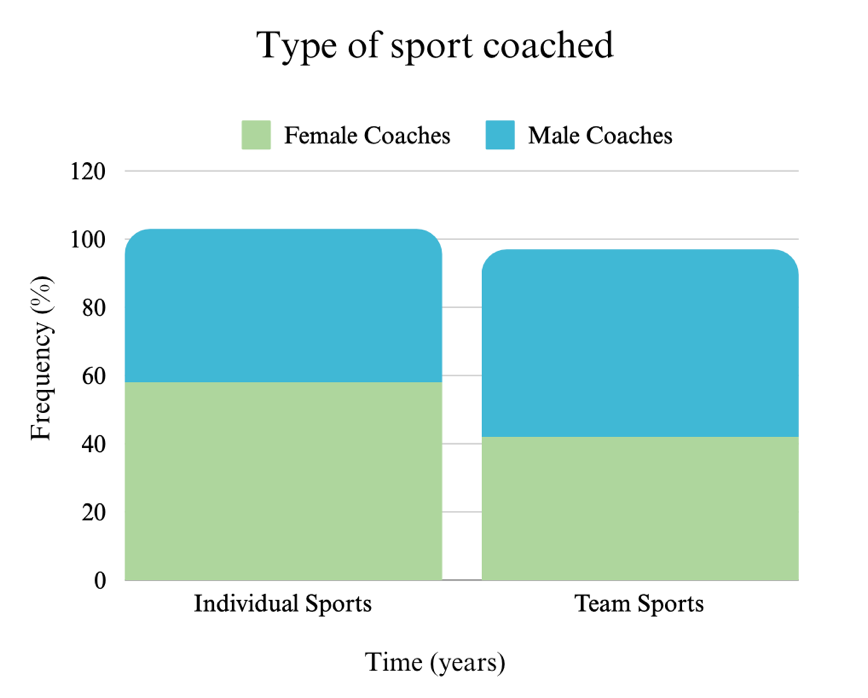


Figure 10 - Frequency distribution of the type of sport coached.
